# Variations in Plasma Membrane Topography Can Explain Heterogenous Diffusion Coefficients Obtained by Fluorescence Correlation Spectroscopy

**DOI:** 10.3389/fcell.2020.00767

**Published:** 2020-08-11

**Authors:** Astrid Gesper, Stefan Wennmalm, Philipp Hagemann, Sven-Göran Eriksson, Patrick Happel, Ingela Parmryd

**Affiliations:** ^1^RUBION, Ruhr-Universität Bochum, Bochum, Germany; ^2^SciLifeLab, Royal Institute of Technology, Stockholm, Sweden; ^3^Institute of Biomedicine, University of Gothenburg, Gothenburg, Sweden

**Keywords:** diffusion, fluorescence correlation spectroscopy, membrane topography, plasma membrane, scanning ion conductance microscopy

## Abstract

Fluorescence correlation spectroscopy (FCS) is frequently used to study diffusion in cell membranes, primarily the plasma membrane. The diffusion coefficients reported in the plasma membrane of the same cell type and even within single cells typically display a large spread. We have investigated whether this spread can be explained by variations in membrane topography throughout the cell surface, that changes the amount of membrane in the FCS focal volume at different locations. Using FCS, we found that diffusion of the membrane dye DiI in the apical plasma membrane was consistently faster above the nucleus than above the cytoplasm. Using live cell scanning ion conductance microscopy (SICM) to obtain a topography map of the cell surface, we demonstrate that cell surface roughness is unevenly distributed with the plasma membrane above the nucleus being the smoothest, suggesting that the difference in diffusion observed in FCS is related to membrane topography. FCS modeled on simulated diffusion in cell surfaces obtained by SICM was consistent with the FCS data from live cells and demonstrated that topography variations can cause the appearance of anomalous diffusion in FCS measurements. Furthermore, we found that variations in the amount of the membrane marker DiD, a proxy for the membrane, but not the transmembrane protein TCRζ or the lipid-anchored protein Lck, in the FCS focal volume were related to variations in diffusion times at different positions in the plasma membrane. This relationship was seen at different positions both at the apical cell and basal cell sides. We conclude that it is crucial to consider variations in topography in the interpretation of FCS results from membranes.

## Introduction

In diffusion studies of membrane molecules with optical techniques, a common assumption is that the membrane is both flat and aligned with the imaging plane. This is a reasonable assumption for supported lipid bilayers (Machan and Hof, 2010), but highly unlikely for biological membranes. The wealth of images from cytoskeletal studies do not show flat cell surfaces and an assumption of flat cell membranes is both surprising and somewhat difficult to justify. Although rarely acknowledged in studies on cells, it has been demonstrated that the global curvature of a membrane will affect diffusion measurements (Holyst et al., 1999; Faraudo, 2002). How we perceive cellular processes like cell signaling, cell adhesion and molecular clustering is also affected by membrane topography (Owen et al., 2013; Parmryd and Onfelt, 2013; Dinic et al., 2015; Jung et al., 2016; Cai et al., 2017).

In the analysis of data from widespread methods employed for diffusion studies of membrane components like fluorescence correlation spectroscopy (FCS), fluorescence recovery after photobleaching (FRAP), and single particle tracking (SPT) it is still assumed that the membrane is locally flat and aligned with the imaging plane. The associated data underpins elaborate membrane models including hop diffusion (Fujiwara et al., 2002), transient anchorage (Chen et al., 2006), and fixed obstacles (Nicolau et al., 2007) to explain non-Brownian diffusion. While it is possible that membrane organization might correspond to these models, non-flat surfaces alone can create the appearance of anomalous diffusion – something we have previously demonstrated for SPT-analysis (Adler et al., 2010, 2019).

**FIGURE 1:**
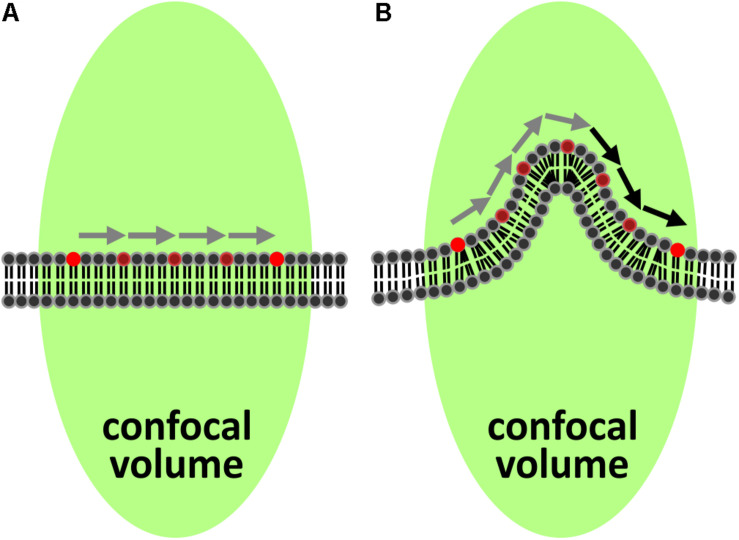
Impact of curved membrane sections on FCS recordings. **(A)** The most common model for analyzing diffusion in two dimensions with FCS assumes a locally flat membrane aligned perpendicular to the optical axis of the excitation beam (green elongated spot). Gray arrows indicate the length of the path from one side of the excitation spot to the other. **(B)** If the membrane is locally curved, the path becomes longer (indicated by the additional black arrows) due to the surplus of membrane within the excitation spot, which leads to longer transit times determined by FCS.

In FCS, fluctuations of fluorescence in a focal volume are analyzed and provide information about several parameters including the diffusion coefficient. When FCS is performed in a membrane, the membrane must be flat and perpendicular to the excitation light (Figure 1A), for the unmodified and commonly used autocorrelation function to correctly describe the data (Malchus and Weiss, 2010). Whenever these criteria are not met, the rate of diffusion is underestimated since the molecules in the membrane may have traveled much further than assumed while remaining in the focal volume (Figure 1B). This has been demonstrated in simulations of folded membranes mimicking the highly convoluted ER and Golgi apparatus (Weiss et al., 2003). Importantly, variations in topography in combination with cells being dynamic could help explain the generally big spread of FCS-measurements for the same cell type.

Scanning ion conductance microscopy (SICM; Hansma et al., 1989) is a non-contact surface scanning technique suitable for imaging the topography of live cells, as reviewed in Happel et al. (2012). In an extensive study covering a wide range of cell types examined live, it was demonstrated that all cell types had extensive topography with ridges, undulations and projections and none could be described as flat (Adler et al., 2010).

More recent developments of FCS including imaging FCS (Krieger et al., 2015) and stimulated emission depletion (STED)-FCS (Mueller et al., 2011) usually are performed at the basal side of cells, i.e., the side in contact with the coverslip. Although the basal side of cell is more restricted in movement than its apical side, topography variations still exist as supported by electron microscopy (Andrews et al., 2008), reflection light microscopy (Barr and Bunnell, 2009), and variable-angle TIRF microscopy (Cardoso Dos Santos et al., 2016).

In this study, we used FCS to examine the diffusion and SICM to assess the topography in different parts of the plasma membrane and analyzed the relationship between the two. For this a method to assess the distribution of membrane topographical features was developed. Performing complementary diffusion simulations and FCS modeling, we show how topography rather than anomalous diffusion can explain longer transit times.

## Materials and Methods

### Materials

Glutamine, DMEM, trypsin-EDTA, and penicillin/streptomycin were obtained from GE Healthcare HyClone (Logan, UT, United States). Fetal bovine serum, enzymes, 3-aminopropyltriethoxy silane (TESPA), L-15 and chemicals were from Sigma (St. Louis, MO, United States). DiI-C_12_, DiD-C_18_, Lipofectamine 2000 and Gibco Opti-MEM were from Thermo Fisher Scientific (Waltham, MA, United States). High performance, 1.5H, coverslips were from Marienfeld (Lauda-Königshofen, Germany). The colon cancer cell line HT29 was a kind gift of Dr. A. Blokzijl, Uppsala University, Sweden and SW480 cells were from ATCC (Manassas, VA, United States). CD3ζ-EYFP, mouse CD3ζ fused to EYFP via a six aa linker expressed in the pBJ1-Neo plasmid under the CMV-promotor, was from Mark Davis, Stanford University School of Medicine, United States (Krummel et al., 2000). Lck-EGFP, a fusion protein of mouse Lck and EGFP via a six aa linker expressed in the pcDNA3 under the CMV-promotor, was from Tony Magee, Imperial College London, United Kingdom (Janes et al., 1999). Borosilicate glass (1B100F-4) was from World Precision instruments (Sarasota, FL, United States).

### Cell Culture

HT29 and SW480 human colon adenocarcinoma cells were cultured in DMEM supplemented with 10% FCS, 100 U/ml penicillin, 100 μg/ml streptomycin, and 2 mM glutamine. The cells were maintained at 37°C in a humidified incubator under 5% CO_2_.

### Transfection of Cells

Cells at 40% confluence plated on TESPA-coated 1.5H coverslips mounted in Petri dishes were washed in DMEM supplemented with 10% fetal calf serum. The cells were transfected with CD3ζ-EYFP or Lck-EGFP using Lipofectamin 2000 according to the instructions of the manufacturer.

### Cell Staining for FCS

Cells were plated on TESPA-coated No. 1.5 high precision coverslips mounted in Petri dishes 34–40 h before imaging. The cells were washed twice with DMEM and stained with 200 μl 2.5 μg/ml DiD-C_18_ at 37°C for 15 min or 1 ml 2.5 μg/ml DiI-C_12_ at 37°C for 15 min or on ice for 5 min, followed by two washes in DMEM without phenol red. Cells stained on ice were kept on ice until the start of the imaging session.

### FCS Setup

Fluorescence correlation spectroscopy measurements were performed on a Zeiss 780 confocal laser scanning microscope equipped for FCS, with a Zeiss water immersion objective, C-Apochromat 40×/1.2 NA. Samples containing DiI were excited at 514 nm or 561 nm and emission was collected between 520–695 nm or 570–695 nm, respectively. Samples containing DiD were excited at 633 nm and emission was collected between 640–695 nm. The 514 nm focus had a beam waist (ω) of 0.22 μm and a volume (V) of 0.36 fl [from measurement of Rhodamine 6G that has a diffusion coefficient (D) of 390 μm^2^/s (Müller et al., 2008) and adjusted for 22°C, yielding τ_D_ = 30 μs]. The 561 nm focus had ω = 0.27 μm and V = 0.64 fl (from measurement of Alexa 568 that has D ≈ 400 μm^2^/s, yielding τ_D_ = 45 μs). The 633 nm focus had τ_D_ = 65 μs, ω = 0.31 μm, and V = 0.71 fl [from measurement of HiLyte 647 that has D = 296 μm^2^/s (Wennmalm et al., 2015) and adjusted for 22°C, yielding τ_D_ = 65 μs]. The 488 nm focus had ω = 0.23 μm and V = 0.36 fl [from measurements of Alexa 488 that has D = 390 μm^2^/s (Petrasek and Schwille, 2008) and adjusted for 22°C, yielding τ_D_ = 32 μs].

### FCS Measurements

Imaging was performed in 2 ml DMEM without phenol red supplemented with 16 U/ml glucose oxidase and 7000 U/ml catalase. The *z*-position for FCS measurements at the top of the cell, i.e., above the nucleus, was set in imaging mode such that the top of the cell was in the focal plane. This yielded the same result as optimizing the *z*-position for maximum fluorescence intensity during FCS-recording. FCS was recorded during 60–120 s at each position; (1) above the nucleus, (2) above the cytoplasm, i.e., midway between the nucleus and the longest axes of the cell spread on the apical cell side, and (3) at varying positions at the basal cell side. The *z*-position for FCS measurements at the apical cell side midway between the nucleus and the longest axes of the cell spread (above cytoplasm) was set in imaging mode such that the in focus-part of the cell, i.e., the position where FCS was recorded, appeared as a stripe around the upper part of the cell in the center part of the image. In order to verify that any difference in τ_D_ between the top of the nucleus and above the cytoplasm measurements was not due to false *z*-positioning during the measurements at the latter, a series of control measurements at this position was performed 0.5 μm above and 0.5 μm below the focus of the plasma membrane, as well as in focus.

### FCS Analysis

Fluorescence correlation spectroscopy curves were fitted using the Zeiss Zen 2012 software to a model for 2D diffusion of a single diffusion component, without triplet. Only the part of the FCS curve slower than τ = 102 μs was fitted in order to avoid the influence of any blinking processes. In a few cases the fitting indicated the need for a model with more than one diffusion component, and τ_D_ was then estimated from the half-amplitude of the FCS curve. We chose not to calculate the weighted mean τ_D_ after fitting to a two-component model because with this approach, in cases when the slower component is very long (several hundreds of ms), the slower component completely dominates the weighted mean τ_D_, even when its relative amplitude is small. The initial value of the fluorescence intensity of DiD, DiO, EGFP, and EYFP was estimated from the intensity trace at the start of the FCS measurement, with an error of ±$\sqrt{I}/2$. For each measurement point, shorter FCS recordings of 4–10 s were repeated to obtain a total measurement time of 60–120 s. Individual 4–10 s measurements were excluded from the analysis if membrane movement or initial photobleaching was present, since these processes distort the FCS curves.

### SICM Measurements

The SICM was a home-build setup previously described (Gesper et al., 2017). In brief, the scanning pipette was mounted on a three-way piezo cube (Nanocube, Physik Instrumente, Germany), which was additionally equipped with a stiff shear force piezo (P-111.05, PI Ceramics, Germany) to allow for faster recording. Glass capillaries were made from borosilicate glass with access resistances between 80 and 120 MOhm and filled with Leibovitz (L-15) medium. The estimated pipette inner radii were 22–32 nm. Assuming a half cone opening angle of 7° typical for pipettes pulled with the setting used (Gesper et al., 2017), these pipettes allow a lateral resolution between 66–96 nm. The cells were scanned in L-15 medium. The approach velocity was 100 nm ms^–1^, the pixelsize 125 nm and the threshold was set to 1%. From scans of flat surfaces (Gesper et al., 2017), we estimate the vertical accuracy of the SICM we used to be in the range of 10 nm.

### Analysis of Cell Surface Roughness

Scanning ion conductance microscopy raw data was filtered using a 3 × 3 pixel median filter. Binary masks for each cell were drawn manually to prevent the overlap of masks from neighboring cells. The mask was applied to the slope data of the scan and convolved with a 2D-Gaussian profile with a full width at half maximum (FWHM) of eight pixels and the height was reconstructed to obtain smoothed height data. The coordinates of the highest point of the smoothed height data were selected as the reference point. Using the slope instead of the height directly avoids the impact of inaccuracies in the positioning of the pipette along the slow scanning direction. However, in some cases where a cell was located next to a second cell, this procedure failed. In that case, the height was convolved directly and data containing notable inaccuracies in the positioning of the pipette along the slow scanning direction were omitted from the analysis. The Euclidean distance from every pixel to the reference point was computed and the maximum distance obtained was used to split the pixels into ten groups. The first group comprised pixels with a distance between 0 and 10% of the maximum distance, the second group pixels with a distance between 10 and 20% of the maximum distance and so on. For display, large artifactual topographical features such as tilt of the cell culture dish or steps along the fast scanning direction, most likely introduced by vibrations when retracting the pipette quickly by a large distance at the end of a line, were removed by linewise fitting of a line to a manually selected region of the data and subsequent subtraction of this line from the data. Roughness was calculated from the processed SICM data only along the fast scanning direction to remove the potential impact of inaccuracies in the positioning of the scanning pipette along the slow scanning direction. Seven consecutive pixels (1–7, 2–8, 3–9, …) along the fast scanning direction were chosen. To remove the general, low-frequency cell shape, a polynomial of fifth degree was fitted to and subsequently subtracted from the data. We then set the roughness of the central, i.e., the fourth, pixel as the root of the mean of the squared deviations from the seven data points.

### Simulations and FCS-Modeling

All simulations and FCS-modeling were performed in Matlab, versions 2017a to 2019b. The diffusion of molecules undergoing Brownian diffusion within a convoluted surface was simulated using an algorithm that enables simulation of diffusion in periodic, nodal surfaces (Wohland et al., 2001), and can operate on any surface defined by the function $\varphi \,\left(\vec{r}\right)=0$. This function was defined by interpolating the data points by cubic splines with the Matlab class *griddedInterpolant*, providing a function *z*(*x*,*y*) that returns the *z*-coordinate for any (*x*, *y*)-coordinate on the interpolated surface.

The gradients ∇⁡*z*(*x*,*y*) along *x*- and *y*-directions of an interpolated set of data points with a pixel size of about 5 nm were computed for every pixel of the interpolated data. The simulation started at a random point $\vec{r_{0}}$ with the lateral coordinates (*x*_0_, *y*_0_) drawn from a uniform distribution. The lateral coordinates of the next point, $\vec{r_{1}}$, were computed as $x_{1}=\cos{\left(\nabla z\left(x_{0}^{\prime },y_{0}^{\prime }\right)\right)}_{x}\Delta x_{0}+x_{0}$ and $y_{1}=\cos{\left(\nabla z\left(x_{0}^{\prime },y_{0}^{\prime }\right)\right)}_{y}\Delta y_{0}+y_{0}$. Here, $\left(x_{0}^{\prime },y_{0}^{\prime }\right)$ indicate the coordinates of a point closest to (*x*_0_,*y*_0_) that lies on the grid of the previously interpolated data points, $\nabla z\,{\left(x_{0}^{\prime },y_{0}^{\prime }\right)}_{x}$ indicates the gradient along the *x*-direction at this data point and Δ*x*_0_ indicates a random length drawn from a normal distribution with mean μ = 0 and standard deviation $\sigma =\sqrt{2\,D\,\Delta \,t}$ where *D* is the diffusion coefficient and Δ*t* the time of one step in the random movement (analogously for *y*). This implements random movement within the tangential plane at $\left(x_{0}^{\prime },y_{0}^{\prime }\right)$ with a length of $\sqrt{\Delta \,x_{0}^{2}+\Delta \,y_{0}^{2}}$. The corresponding *z*-coordinate was then computed by projecting the point back onto the surface. The procedure was repeated for $\vec{r_{2}}$ by using $\vec{r_{1}}$ as the previous data point, etc.

The intensities of the simulated fluorescent molecules were calculated using a hypothetical confocal microscope (Qian and Elson, 1991; Holyst et al., 1999; Wohland et al., 2001) with the following optical parameters and the corresponding notations: λ_Exc_ = 570 nm, wavelength of the excitation light; λ_Em_ = 620 nm, wavelength of the emitted light; *n* = 1.518, refractive index of the medium; NA = *n* sin α = 1.4, numerical aperture of the objective, with α denoting the half cone angle of the objective; *M* = 1, magnification of the objective; *R*_ph_ = 0.5 AU, pinhole radius in units of the Airy disk diameter (AU). Note that a magnification of *M* = 1 was used to simplify the calculation of the effect of the pinhole. Calculations were restricted to components that have a position-dependent effect on the intensity such as the confocal pinhole, but omitted photophysical relations such as quantum yield.

The excitation beam was positioned in the center of the investigated area and its *z*-position was selected as the *z*-coordinate of the respective pixel. In the following, we use the index *S* to indicate coordinates with respect to the excitation beam. The intensity *I* of a molecule at position $\vec{r_{S}}=\left(x_{S},y_{S},z_{S}\right)$ was calculated as $I\,\left(\vec{r_{S}}\right)=\gamma \,\left(\vec{r_{S}}\right)\,\rho \,\left(\vec{r_{S}}\right)$. Here, $\rho \,\left(\vec{r_{S}}\right)$ describes the effect of the pinhole and $\gamma \,\left(\vec{r_{S}}\right)$ the intensity distribution of the focused laser beam. The latter was approximated as a three-dimensional Gaussian distribution: $\gamma \,\left(\vec{r_{S}}\right)={\left(w_{0}\,w_{z}^{-1}\right)}^{2}\,\exp\left(-2\,\left(x_{S}^{2}+y_{S}^{2}\right)\,w_{z}^{-2}\right)$. Here, *w*_0_ = 0.5(Γ/(2*ln*⁡(2)))^1/2^ is the radius of the beam at intensity *exp*⁡(−2) with Γ = λ_*Exc*_(2NA)^−1^ denoting the FWHM of the diffraction limited beam; *w*_*z*_ = *w*_0_(1 + (*z*_*S*_/*z*_0_)^2^) describes the width of the beam as a function of the *z*-coordinate with $z_{0}=\pi \,w_{0}^{2}/\lambda_{E\,x\,c}$ denoting the Rayleigh length of the diffraction limited beam. Note that in practice, *z*_0_ often is larger due to compromises between signal strength and resolution.

The effect of the pinhole was calculated by the following geometrical considerations: The amount of light emitted from a fluorophore and collected by the objective is limited by the half cone angle α of the objective. Hence, the radius *R*_z_ of the cone from a molecule located at an arbitrary *z*_S_ position is *R*_z_ = *R*_0_ + *z*_S_ tan α. Here, *R*_0_ is the minimum radius of the cone, which was defined as the resolution limit of the microscope *R*_0_ = λ_*Em*_(2NA)^−1^. It was assumed that all light that passes the pinhole is detected, hence, the effect of the pinhole can be calculated by determining the area of intersection *A* between the pinhole and the cone of light emitted from the molecule. For this, let $d_{S}={\left(x_{S}^{2}+y_{S}^{2}\right)}^{1/2}$ denote the distance from the molecule to the optical axis. Due to the symmetry of the system, it is sufficient to consider molecules at positions (*d*_S_ ≥ 0, *z*_S_ ≥ 0). If the distance *d*_S_ of the molecule to the center of the pinhole is larger than the sum of the pinhole radius *R*_ph_ and the cone radius, the overlapping area *A* = 0. If *R*_ph_ ≥ *R*_z_ and *R*_z_ + *r*_S_ ≤ *R*_ph_, all emitted light passes the pinhole, hence, $A=\pi \,R_{z}^{2}$. If *r*_S_ − *R*_z_ ≤ −*R*_ph_, the pinhole is completely covered by light, hence, $A=\pi \,R_{\text{ph}}^{2}$. In all other cases, the pinhole and the cone intersect and we computed the area of intersection ζ of the corresponding circles, hence A = ζ (*R*_ph_, *R*_z_, *r*_S_). The effect of the pinhole ρ was computed as $\rho \,\left(\vec{r_{S}}\right)=A\,{\left(\pi \,R_{z}^{2}\right)}^{-1}$. To compute the auto-correlation G(τ) of the sum of the intensities of the single molecules after one simulation step, an adaption of the Matlab multiple *tau* algorithm, that can be found at https://pypi.python.org/pypi/multipletau/, was used. A single-spot FCS model *G*(τ) = *N*^−1^(1 + (τ/τ_*D*_))^−1^ was fitted to the autocorrelation data. Here, *N* is the average number of molecules located in the focus of the excitation beam and τ_D_ is the average transit time of molecules diffusing through the focus. Fits were performed using Matlab’s fit function from the Curve Fitting Toolbox, which implements a linear least squares algorithm.

To obtain the transit times at different positions of the cell membrane, the free diffusion of molecules with a density of ∼10 molecules/μm^2^ were simulated five times. The diffusion coefficient used was 0.1 μm^2^/Δ*t* and Δ*t* was selected as 10^–3^ ms. At this readout time, these settings correspond to a diffusion coefficient of 0.1 μm^2^/s, a typical diffusion coefficient for plasma membrane proteins (Chojnacki et al., 2017). In a perfectly flat and smooth membrane, this procedure yields transit times approximately 1% larger than expected from the simulated diffusion coefficient with a standard deviation of about 10%.

The selection of positions for diffusion simulations and FCS modeling was arbitrary and only cell surfaces were included. In addition, it was ensured that the corresponding membrane regions did not overlap and thus represented different parts of the plasma membrane.

### Assessment of Anomalous Diffusion

To assess apparent anomalous diffusion, FCS was modeled at 14 arbitrarily selected positions at five excitation spot sizes. The FWHM ranged from ∼65 nm, which corresponds to spot sizes obtained by STED-FCS, to ∼300 nm, which corresponds to FCS in the near infrared region. The spot sizes are reported as multiples of the area of the modeled beam described in section “Simulations and FCS-modeling” as used for the previous FCS modeling, referred to as the reference spot size. The model *A* × *s*^α^ was fitted to the mean transit times for the five simulations at each position and spot size. To account for the different uncertainties in the transit time, means for the different spot sizes were weighted by their inverse variance multiplied by the respective mean. In the model, *s* indicates the size of the excitation spot and *A* a free scaling factor. For free Brownian diffusion, the relationship between transit time and spot size is directly proportional with α = 1. Any deviation of α from 1 indicates anomalous diffusion and in our simulations; the smaller the α the larger the apparent anomalous diffusion. To assess whether the degree of apparent anomalous diffusion was related to the transit time of the reference spot size, the Pearson correlation coefficient between α and the transit time was used.

### Statistical Analysis

The τ_D_-values from FCS were compared using a paired, two-sided *t*-test. Using the Anderson–Darling test, it could not be rejected that the data were normally distributed. The roughness was analyzed by linear ANOVA models using the mean of the 90th percentiles of the 10 distance groups of all investigated cells and *t*-tests were performed on log_10_-transformed values. For the comparisons of the means of the 90th percentile, a pairwise *t*-test including the Holm-Bonferroni method to correct for multiple comparisons was used. Whether τ_D_-values could be explained by *I*-values was assessed using multiple linear regression analysis after log_10_-transformation of the τ_D_ and *I* values to make the data less skewed. The effect the variables log_10_(I_p__rotein_), log_10_(I_DiD_), cell type, protein type, positions for the FCS-measurements and individual cells on log_10_(τ_D_) were estimated simultaneously with least squares in a multiple linear regression model with the restriction that the slope was the same for all experiments. In the model all observations were given equal weight.

## Results

Cells are never smooth and flat, which we have shown dramatically affects the interpretation of SPT data (Adler et al., 2010, 2019). FCS of membrane components would appear to be equally prone to topographical artifacts and this we set out to investigate.

### Diffusion in the Plasma Membrane Appears to Be Faster Above the Nucleus Than Above the Cytoplasm

**FIGURE 2:**
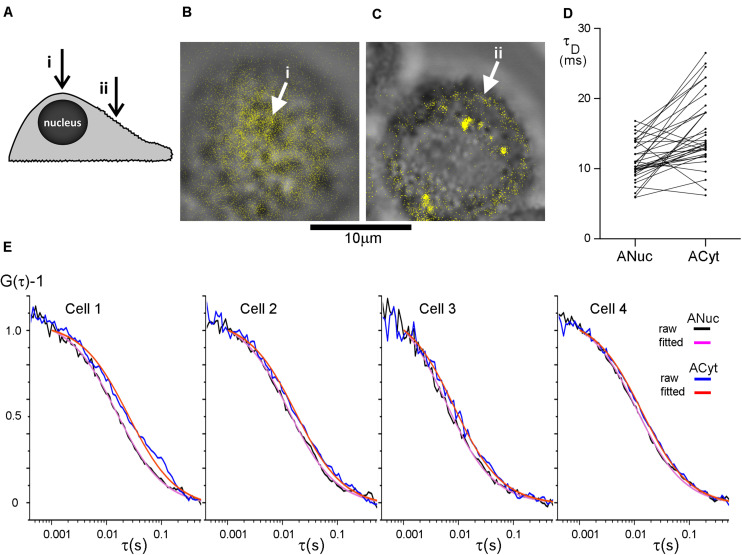
The diffusion in the plasma membrane appears to be faster on top of the nucleus than over the center of the cytoplasm. **(A)** The cells were subjected to FCS measurements at two different positions at their apical side plasma membranes; (i) on top of the nucleus and (ii) midway between the nucleus and the longest axes of the cell spread, above the cytoplasm center (indicated with arrows). HT29 cells stained with DiI imaged at **(B)** the top of the nucleus and **(C)** over the center of the cytoplasm with (i) and (ii) corresponding to panel **(A)**. The DiI-signal in panels **(B,C)** was intensity thresholded and is displayed in false color. Scale bar 10 μm. **(D)** Comparison of the difference in τ_D_ from the plasma membrane on top of the nucleus and over the center of the cytoplasm. *p* = 5.0 × 10^–6^ for a two-tailed, paired *t*-test, and *n* = 36. Values from one cell were excluded from the graph, but not from the analysis, for clarity because its τ_D−_values were considerably longer. **(E)** FCS curves from four different HT29 cells. On each cell FCS was measured above the nucleus (experimental curve in black, fitted curve in purple) and over the center of the cytoplasm (experimental curve in blue, fitted curve in red). Curves were fitted to a model assuming one diffusing species starting the fit at τ = 102 μs.

In FCS, a considerable spread of data from diffusion coefficient measurement of plasma membrane components is commonplace (Ries and Schwille, 2008; Stromqvist et al., 2011). We suspected that variations in the positioning of the excitation beam at the apical side of the plasma membrane could help explaining this variation. To this end we used human colon cancer HT29 cells, an adherent epithelial cell type, that maintain a rounded shape when cultivated on glass or plastic rather than becoming extensively stretched. Epithelial cells, like many other cell types, are rich in topographical features (Adler et al., 2010), but the distribution of these features have not been well characterized. We decided to compare whether there was a difference in diffusion on top of the nucleus and over the center of the cytoplasm (Figure 2A). Measurements above the nucleus were performed at the top of the cell (Figure 2B) and measurements above the cytoplasm were performed midway between the nucleus and the longest axes of the cell spread. When in focus, this area of the plasma membrane appears as a fluorescent band around the upper part of the cell and this band was positioned in the center part of the image before the FCS measurements (Figure 2C). This focusing method allowed us to check after each completed FCS measurement that the plasma membrane remained in focus.

Confocal FCS was used to study the diffusion of DiI-C_12_ in the plasma membrane of HT29 cells. A comparison of the correlation time, τ_D_, which is inversely proportional to the diffusion coefficient, suggested that the diffusion in the plasma membrane on top of the nucleus was faster than that over the cytoplasm. Interestingly, minimizing the risk of the internalization of the membrane probe by labeling cells on ice, with the FCS measurements performed immediately after labeling at room temperature, resulted in a similar trend as labeling at 37°C. Therefore, results from both labeling temperatures were pooled in the analysis. The absolute values of τ_D_ varied considerably from cell to cell, but the difference in τ_D_ between the two positions was highly significant with τ_D_ over the cytoplasm being longer than τ_D_ on top of the nucleus (Figure 2D). The difference in τ_D_ at the two positions is illustrated for four individual cells, fitted with a model for 2D diffusion of a single diffusion component (Figure 2E).

To verify that the observed difference in τ_D_ above the nucleus and above the cytoplasm was not due to incorrect *z*-positioning during the measurements, control measurements 0.5 μm below and above the fluorescence band, i.e., focus in the cytoplasm and outside the cell, respectively, were performed. These measurements confirmed that a one-component-model was appropriate and that false positioning was not causing the difference in τ_D_ for the two positions. The measurements 0.5 μm above and below focus did not differ significantly from measurements of the plasma membrane in focus, i.e., incorrect *z*-positioning was unlikely to explain the longer τ_D_ observed above the cytoplasm as compared to that above the nucleus. If we inadvertently had assigned 0.5 μm above the PM as the in focus position, then defocusing by ±0.5 μm should have yielded a 50% increase in τ_D_ for the +1.0 μm position, and a 20–25% decrease for the 0.0 μm position (Humpolickova et al., 2006).

### Cell Surface Roughness Is Unevenly Distributed

**FIGURE 3:**
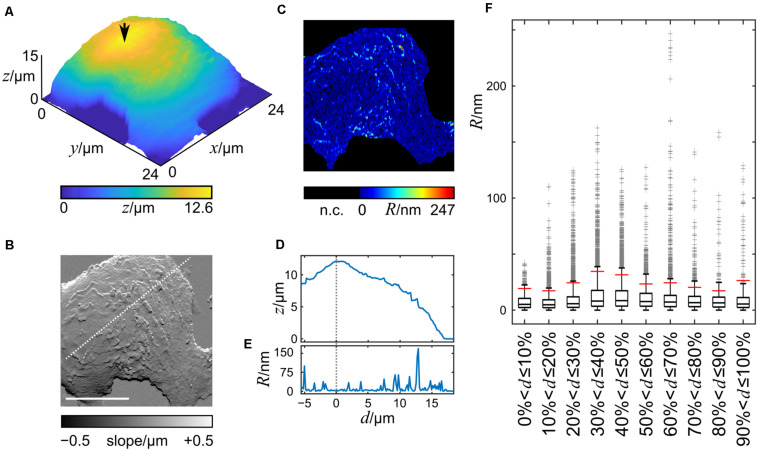
3D-Height and slope representation of a section of a living HT29 cell imaged by SICM. Height and slope representation of a HT29 cell imaged live by SICM is shown in **(A,B)**. **(C)** Computed roughness of the cell surface. The cell chamber bottom and the steep transitions between the dish and the cell were not considered (n.c.). The arrow in A indicates the position of the reference point. Scale bar in B: 10 μm, also applies to **(C)**. **(D)** Height and **(E)** roughness profiles along the dashed white line shown in **(B)**. The vertical dashed line in **(D,E)** represents the position of the highest point of the nucleus. **(F)** Box plot of the roughness data grouped into 10 equally spaced groups with increasing distance from the reference point. Boxes indicate the lower and upper middle quartile, whiskers data within ± 1.5 inter quartile ranges, gray crosses indicate outliers.

To investigate whether the topographic features are inhomogeneously distributed across the cell surface, we imaged living HT29 cells by SICM, one cell measurement is shown in Figure 3A. The maximum cell height observed was 12.6 μm and the cell footprint was larger than the scanning frame of 24 × 24 μm. In the slope representation topographic features of the cell membrane are visible (Figure 3B), with single protrusions extending approximately 1 μm from the cell’s silhouette (Supplementary Figure 1). The roughness, calculated as the deviation from the mean height of a 7 × 1 pixel window (875 × 125 nm), after subtracting the cell silhouette, ranged from 0 nm to 247 nm with average of 14.9 ± 38.4 nm and the data were heavily skewed toward higher values (Figure 3C). Under these imaging conditions, the roughness of the coated cell culture dish was between 0 and 5 nm.

A profile of the roughness along a line (Figure 3B) through a reference point, selected as the highest point of the cell after smoothing (indicated by the arrow in Figure 3A), shows that the roughness is not evenly distributed (Figures 3D,E). The lowest cell surface roughness is found at the highest part of the cells.

To test whether our finding could be generalized to the entire cell surface, we split the cell surface into ten groups of pixels, the first group comprising pixels with an Euclidean distance between 0 and 10% of the maximum distance from the reference point, the second group comprising pixels between 10% and 20% of the maximum distance, etc. Figure 3F shows a box plot of the roughness values of the cell membrane using this grouping. A large number of points exceeded 1.5 inter quartile ranges (IQRs) indicating that the data is skewed toward higher values. Consequently, the difference of the medians was small with the lowest median at 5.0 nm (second group) and the highest at 8.5 nm (fifth group) while the difference of the maxima of the groups was large with values from 41.9 nm (first group) to 247.0 nm (seventh group). The minima of all groups were 0.0 nm.

Since we wanted to test whether extreme roughness values, i.e., the larger topographical features, are distributed inhomogenously across the cell, we chose to investigate the 90th percentile of the roughness data, *R*90, indicated by the red vertical bars in Figure 3F.

**FIGURE 4:**
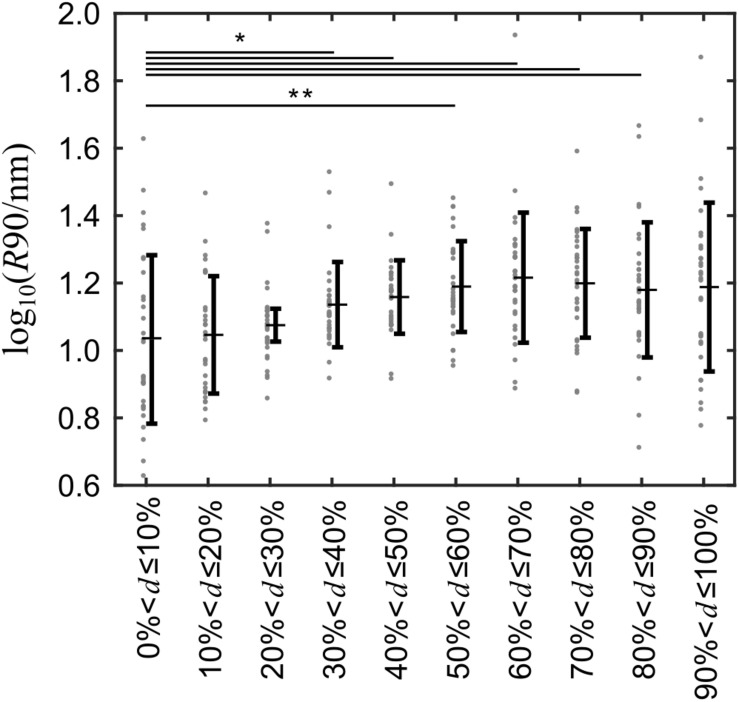
Surface roughness is unevenly distributed over the cell surface and greater away from than at the top of the cells. The cell membrane roughness of 31 different cells was recorded and grouped into 10 evenly spaced groups relative to the spatial dimension of each cell and the log-transformed means of the 90th percentile of the roughness data in each group was compared with a paired *t*-test. Gray dots indicate the 90th percentile of the cell surface roughness for every cell, horizontal bars with whiskers indicate the mean ± standard deviation. *p < 0.05; **p < 0.01 versus Group 1.

The grouping procedure described above was applied to 31 cells (Supplementary Figure 2) and the corresponding *R*90-values were analyzed. The *R*90 means of groups 4–9, i.e., away from the nucleus were significantly larger than that of group 1 at the top of the nucleus (Figure 4). The *p*-values adjusted for multiple comparisons are provided in Supplementary Table 1.

### FCS Modeled on Simulated Diffusion in Experimentally Obtained Cell Surface Support FCS Data From Live Cells

**FIGURE 5:**
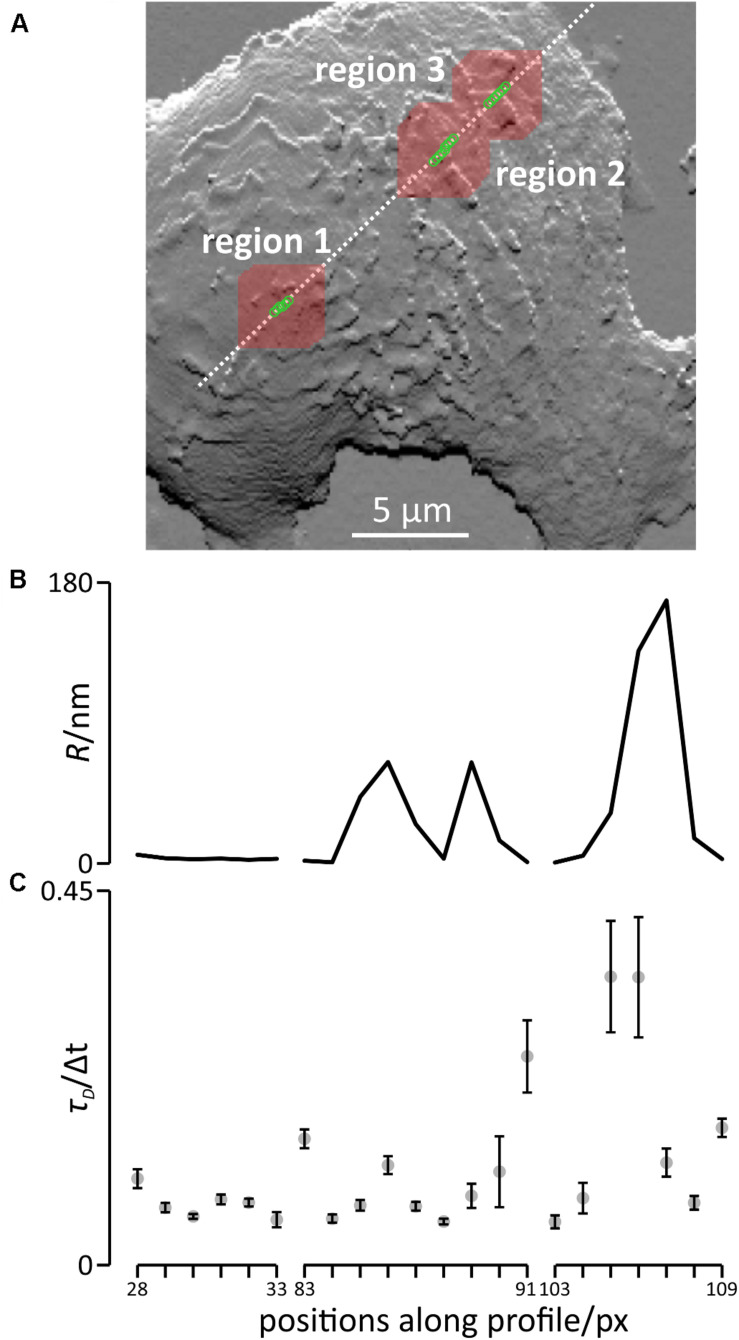
Simulation of diffusion and modeled FCS recordings in the apical part of the membrane of living cells. **(A)** Positions of the modeled FCS recordings (green circles) and areas in which diffusion was simulated (red squares around each green circles) superimposed on the slope plot of the topography of the plasma membrane (Figure 3B). **(B)** Roughness of the cell membrane at the positions of the modeled FCS recordings. **(C)** Transit times presented as means ± SD, *n* = 5.

To investigate whether the inhomogeneous distribution of the topographical features would have an effect on FCS measurements, we simulated free diffusion in the sections of the cell shown in Figure 3A and modeled FCS-recordings at three different regions along the profile shown in Figures 3B–D. The respective regions are highlighted in Figure 5A, the red areas indicate the sections of the cell in which diffusion was simulated. The first region was located at the highest point of the cell and comprised a smooth area of the plasma membrane, the second region comprised an area of the plasma membrane containing two sections of higher roughness located in close proximity, and the third region comprised an area of the plasma membrane containing the maximum roughness observed along the profile (Figure 5B).

The results of the modeled FCS recordings are shown in Figure 5C and the corresponding auto-correlation curves are shown in Supplementary Figure 3. Note that we provide the resulting correlation times, τ_D_, in units of Δ*t*, the time of a single simulation step. In region one, the results of the modeled FCS recording only varied slightly if the position was slightly shifted, yielding transit times ranging from 0.055 ± 0.009 Δ*t* at position 33 to 0.104 ± 0.011 Δ*t* at position 28, a 1.9 fold difference. In region 2, the shortest transit time was 0.052 ± 0.003 Δ*t* at position 88, the longest transit time was 0.251 ± 0.043 Δ*t* (position 91), almost a 4.8 fold difference. In region 3 (bottom panel), the shortest transit time was 0.052 ± 0.008 Δ*t* (position 103), the longest transit time was 0.347 ± 0.067 Δ*t* (position 105), a 6.7 fold difference. In summary, shifting the position slightly has a huge impact in regions rich in topographical features or with high topography differences.

To assess whether even larger differences in the observed transit times could be found, we simulated diffusion and modeled FCS recordings at 14 additional arbitrarily selected positions spread over the cell surface (Supplementary Figure 4). The resulting transit times ranged from 0.040 ± 0.003 Δ*t* to 0.121 ± 0.012 Δ*t.* Overall, the transit times observed for diffusion in this particular cell varied by factor of approximately 6.7. When simulating diffusion on ten arbitrarily selected positions on a second cell using the same parameters, we found a variation by a factor of 3.2 (Supplementary Figure 5).

**FIGURE 6:**
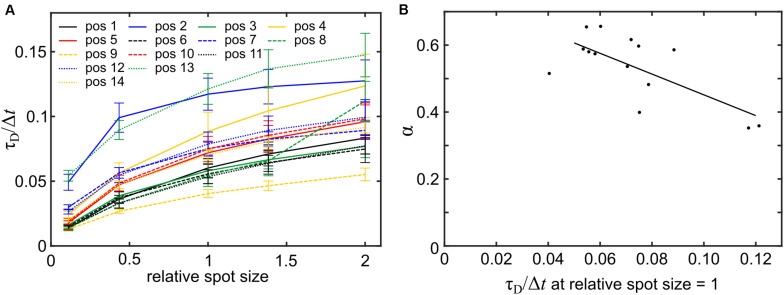
Modeling spot size variation FCS reports topography-induced apparent anomalous diffusion. **(A)** Mean transit times (±SD, *n* = 5) for varying spot sizes. Spot sizes are presented as multiples of the area of the reference spot size (FWHM of ∼200 nm) used in Figure 5C. **(B)** Scatter plot of the parameter α obtained from fitting the model *A* × *s*^α^ to the data in panel **(A)** and the transit time at the reference spot size. The Pearson correlation coefficient for the two variables was −0.70 (*p* = 0.0044).

Next, we assessed whether plasma membrane topography could lead to the erroneous reporting of anomalous diffusion in FCS as it does in SPT (Adler et al., 2010). To this end, spot size variation FCS was modeled at the 14 arbitrarily selected positions shown in Supplementary Figure 4A. At all positions, the relationship between spot size and transit time was non-linear, indicative of anomalous diffusion (Figure 6A). The model *A*×*s*^α^, with *s* representing the excitation spot size, *A* representing the transit time obtained from modeling FCS with the reference spot size and α representing an estimate of the degree of anomalous diffusion was fitted to the data. The α-values ranged from 0.35 ± 0.14 to 0.65 ± 0.08 (errors indicate the 95% confidence interval) and the mean of the α-values was 0.53 ± 0.10 (errors indicate standard deviation). As anticipated, a higher degree of anomalous diffusion was found at positions that showed a higher transit time when modeling FCS at the reference spot size (Figure 6B). In summary, we found that more convoluted membrane sections lead to larger overestimations of the transit times accompanied by a higher degree of apparent anomalous diffusion.

### Variations of the Amount of a Membrane Marker in the FCS Focal Volume Can Explain Variations in τ_D_

**FIGURE 7:**
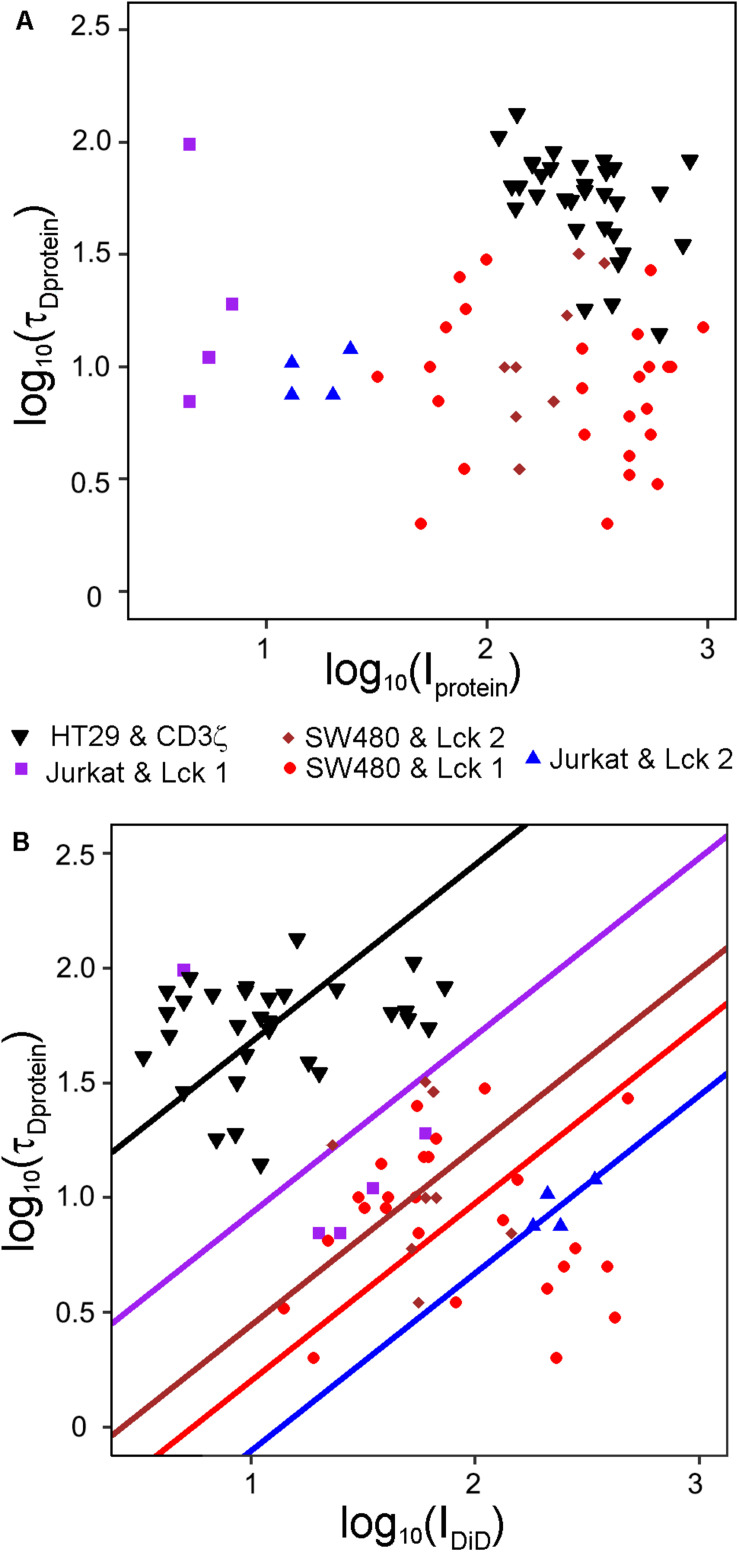
Variations in τ_D_ for plasma membrane proteins can be explained by the membrane density in the focal volume. τ_D_ and I at the start of FCS measurement were determined for DiD-labeled SW480 and Jurkat T cells transfected with Lck-EGFP or HT29 cells transfected with CD3ζ-EYFP. The FCS measurements were conducted in the plasma membrane above the top of the nucleus, above the cytoplasm midway between the top of the nucleus and the cell edge in the direction the cell had stretched the most and at the basal cell side in touch with the coverslip. Curves were fitted to a model assuming one diffusing species starting the fit at τ = 102 μs. Scatter plots of **(A)** log_10_(τ_D_) versus log_10_(I) for the proteins and **(B)** log_10_(τ_D_) for the proteins versus log_10_(I) for DiD. The values from five different experiments are displayed with different symbols. The lines in B were fitted with least squares in a multiple linear regression model where log_10_(τ_D_) was the dependent variable and log_10_ (I_DiD_) was the independent variable. The mean value of log_10_ τ_D_ conditioned by log_10_ I shifted between the different experiments, modeled by indicator variables. Random effects for different cells within each experiment were included in the model, but not in the graph. The model had the restriction that all experiments should have the same slope, which was estimated to be 0.77. The variable log_10_(I_DiD_) was statistically significant (*p* = 0.0092), but log_10_(I_protein_) was not significant (*p* = 0.16) and therefore no lines were fitted in **(A)**. *R*^2^ for the model was 0.83.

Given the topography variations of cells, the amount of membrane in the FCS focal volume will differ, which could explain the large variations in τ_D_ observed for membrane proteins (Stromqvist et al., 2011). Previously, counting of molecules in the FCS focal volume has been used to account for global membrane curvature (Honigmann et al., 2014). We reasoned that the topography variation should be reflected in the fluorescence intensity of a membrane marker at the start of a FCS-measurement and could be used to determine the contribution of topography to the variation in τ_D_ for a membrane protein. To this end, we stained HT29 cells transfected with the transmembrane plasma membrane protein CD3ζ linked to EYFP and SW480 cells or Jurkat T cells transfected with Lck-EGFP. All three cell types were labeled with DiD. We first investigated whether the intensity of the protein constructs themselves could explain the variations in τ_D._ Interestingly, we found that the initial intensities of EGFP and EYFP could not explain variations in τ_D_ for the protein constructs (Figure 7A). Using multiple linear regression analysis, we found a relationship between τ_D_ for the proteins and the initial intensity of DiD (Figure 7B). Neither the position in the plasma membrane at which the FCS measurements were performed, i.e., the bottom of the cells, above the nucleus, or above the center of the cytoplasm, nor the cell or protein type had a statistically significant effect. However, the relationship between τ_D_ and I_DiD_ was as expected not perfect, meaning that not all of the variability in τ_D_ for the two proteins could be accounted for by variations in the amount of membrane in the focal volume.

## Discussion

The plasma membrane is a compartment where several fundamental biological processes take place, e.g., cell adhesion, endo- and exocytosis and signaling via receptors that recognize extracellular ligands. It is therefore important to understand plasma membrane dynamics of which the diffusion modes of its components play a prominent role. FCS is a powerful method for studying diffusion, but in plasma membrane studies it is known to report data with a big spread both within and between cells for the same membrane protein (Schwille et al., 1999; Ries and Schwille, 2008; Stromqvist et al., 2011). Reasons for this include membrane undulations and misalignment of the membrane and the focal volume (Milon et al., 2003; Malchus and Weiss, 2010). Membrane undulations are large-scale membrane fluctuations that result in changes in membrane topography but, importantly, the undulating membrane is considered to be smooth. However, using live cell SICM it has been demonstrated that the plasma membrane is far from smooth (Gorelik et al., 2003; Adler et al., 2010). The topography of the plasma membrane is the result of an interplay between turgor pressure, the cytoskeleton, the plasma membrane and the glycocalix (Shurer et al., 2019). We therefore suspected that local topography variations could play a prominent role in explaining the large spread observed in FCS results.

We show that in HT29 cells diffusion in the plasma membrane as determined by FCS appears to be slower in plasma membrane segments that are located above the cytoplasm than in segments located on top of the nucleus. Using SICM we also found more pronounced topographical features in areas of the plasma membrane not overlying the nucleus in this cell type. Hence, we found that longer transit times coincided with regions richer in topographical features indicating a causal relationship.

To analyze the size and occurrence of topographical features, we transferred a measure used in the material sciences to biology: The surface roughness. Mainly, lamellar structures were observed on the cells investigated by SICM. Lamellar structures are smooth in the direction parallel to their fronts, and abrupt height changes only occur at the front of the lamella. Since the size of the window used to compute the surface roughness was smaller than the observed lamellar structures, high roughness values were only observed at the fronts of the lamellar structures. This was considered by analyzing the 90th percentile of the roughness data after grouping the roughness data according to their distance from the nucleus. We found that the 90th percentile of groups away from the nucleus was significantly higher than the 90th percentile of the group located on top of the nucleus. Importantly, the distribution of topographical features differs from cell to cell emphasizing the importance of establishing the local topography where the FCS measurement is being conducted.

According to the FCS diffusion law, the diffusion behavior of plasma membrane components can be extracted if FCS is performed at different focal volume sizes (Wawrezinieck et al., 2005; Schneider et al., 2018) or *z*-planes (Humpolickova et al., 2006). If the relationship between the focal volume and the transit time deviates from linearity the diffusion is considered anomalous, explained by diffusion barriers or trapping in the form of transient binding, or partitioning to membrane nanodomains (Winkler et al., 2017). Topography is generally not considered as a cause for anomalous diffusion but our results strongly suggest that its consideration is highly relevant as does a recent study (Gupta et al., 2020). As outlined above, the effect of the membrane topography on the transit times is dependent of the FCS focal volume and could therefore account for a non-linear relationship with the measured diffusion coefficient. In particular, considering membrane topography is important when interpreting high-resolution FCS recordings using STED-FCS instruments, where the focal volumes can be reduced to tens of nanometers (Eggeling et al., 2009; Honigmann et al., 2014; Benda et al., 2015; Sezgin et al., 2019), as we showed by modeling spot size variation FCS.

To assess whether differences in experimental FCS data are caused by an uneven distribution of topographical features in the plasma membrane, concurrent recording of both variables would be useful. However, an instrument combining SICM and FCS recordings has yet to be developed, but correlated SICM and fluorescence measurements have been performed, even with super-resolution microscopy methods (Gorelik et al., 2002; Hagemann et al., 2018). An alternative approach is to use the intensity of a membrane marker as a proxy for membrane volume within the FCS focal volume. As we demonstrate, intensity variations of the membrane marker can, to a large extent, but not fully, explain variations in transit times of membrane proteins. This we observed in three different cell lines – two adenocarcinoma colon cancer cell types that are adherent and Jurkat T cells that grow in suspension and for two different proteins – the Src-family lipid anchored Lck and the transmembrane CD3ζ subunit of the T cell receptor. Moreover, the fluorescence intensity in the focal volume could explain differences in transit times regardless of the position in the plasma membrane; the basal side or the apical side either on top of the nucleus or the cytoplasm. Any difference remaining between positions when topography has been accounted for could be due to anomalous diffusion caused by the proteins interacting with other plasma membrane components and/or domains of the plasma membrane like ordered membrane domains. However, the interpretation is complicated by topographical features themselves being able to cause anomalous diffusion both in the form of sub- and superdiffusion (Adler et al., 2019).

The plasma membrane contains two co-existing liquid phases – the more loosely packed liquid disordered (ld) phase and the more tightly packed liquid ordered (lo) phase, of which the latter forms ordered membrane nanodomains known as lipid rafts. A basic requirement for a membrane probe used to report variations in membrane topography is that it distributes randomly in the bulk membrane and co-existing membrane nanodomains and does not show preferential partitioning. Partition studies are generally performed in model membranes with well separated ld- and lo-phases and in those the short acyl chain DiI (C_16_ and lower) probes display a preference for ld-phase whereas for the longer chain DiI (C_18_ and higher) probes the lipid composition determines their phase preference (Baumgart et al., 2007). However, the composition and asymmetry of the plasma membrane are not well represented by these model membranes and the difference between any co-existing phases in the plasma membrane is much smaller (Sezgin et al., 2012; Dinic et al., 2013; Fujimoto and Parmryd, 2016). It is therefore reasonable to assume that the fluorescence intensity of probes used in this study, DiI-C_12_ and DiD-C_18_, are good proxies for the amount of membrane present in the FCS focal volume.

Interestingly, in contrast to DiD-C_18_, the two protein constructs, Lck-EGFP and TCRζ-EYFP, did not prove to be good proxies for the amount of plasma membrane in the focal volume. The reason could be because they both are enriched in ordered membrane nanodomains and hence may not be homogenously distributed (Kabouridis et al., 1997; Dinic et al., 2015). Moreover, neither Lck nor TCRζ are exclusively plasma membrane proteins. Two days past transfection, the protein constructs well represent the distribution of the endogenous proteins, which are both found in intracellular membranes in addition to the plasma membrane. Since the elongated focal volume extends into the cytoplasm, endosomes, and vesicles are likely to be included and cause an overestimation of the fluorescence intensity in the plasma membrane. The diffusion from such vesicles is accounted for in the subsequent FCS analysis, but in practice it is difficult to account for the fraction of the fluorescence originating from the vesicles. The membrane markers, on the other hand, were added to the cells just before the FCS recordings were made and hence the label was primarily found in the plasma membrane.

Theoretical studies have concluded that membrane curvature *per se* will slow down the diffusion of membrane proteins since it influences the packing in the two leaflets and hence the interaction between the protein and the lipids (Gov, 2006; Yoshigaki, 2007; Kabbani et al., 2017). This should also be considered as an explanation for variations in τ_D_, since close to endless combinations of curvature in the focal volume are possible for the same membrane area. In addition, thermal fluctuations, resulting in membrane undulations, may contribute to variations in τ_D_ and even more to the total fluorescence intensity (Reister and Seifert, 2005; Ries and Schwille, 2008; Machan and Hof, 2010).

To estimate the potential impact of the topographical features of the membrane on FCS data, we modeled FCS recordings on these structures with a hypothetical microscope operating at the resolution limit. Under these conditions, we found differences in the transit times of up to sevenfold with slight variations in the position of the excitation beam. However, this number may vary between cells since we found a variation around three in a second cell. In our experimental data, we observed differences of up to threefold in the plasma membrane above the nucleus and cytoplasm for the same cells, in line with the simulations. These variations could be, and probably have been, misinterpreted as anomalous diffusion. It is important to keep in mind that SICM is a surface scanning method and thus better at reporting membrane protrusions than invaginations. Moreover, lateral membrane folding will be missed by SICM. The amount of membrane modeled from the SICM measurements therefore is likely to be an underestimate of the amount of plasma membrane. A reasonable assumption would then be that we should find larger differences in the experimental rather than simulated FCS. However, in practice, resolution is often sacrificed to obtain a better signal-to-noise ratio. A larger focal volume, as used in the FCS experiments compared with the simulations, averages over a larger volume and therefore the impact of vertical topographical features is reduced. This effect we observed when modeling spot size variation FCS based on the SICM data.

By simulating diffusion and modeling FCS recordings, we found a relationship between the plasma membrane roughness and the transit times in FCS recordings supporting the experimental findings. We also showed that an increase in the transit time of up to factor of seven due to differences in membrane topography is possible in FCS recordings at different plasma membrane positions of a single cell. However, we could not use the roughness values to predict the transit times of FCS. There are two reasons for this. Firstly, this is an effect of the window of 7 × 1 pixels, selected to compute the roughness, being smaller than the focal volume modeled to probe the fluorescence intensity. However, we selected this window size since it allowed processing of the data without correcting for inaccuracies along the slow scanning direction, which are a common problem in scanning probe microscopy (Voigtländer, 2015), ensuring that no artificial topographic features are introduced by the correction procedure. Secondly, different topographies can produce the same roughness value, but might lead to different FCS transit times, i.e., the exact topography affects the transit times.

By modeling spot size variation FCS we have shown that topography deviations from a flat and smooth plasma membrane will report anomalous diffusion. Thus FCS, when topography is not considered, can easily be misinterpreted as anomalous diffusion. Our data demonstrate a large cell-to-cell and within cell variation of the plasma membrane topography and hence it needs to be assessed for each cell in order to avoid the over reporting of anomalous diffusion.

In conclusion, we have demonstrated that topography variations can to a large extent explain differences in FCS measurements of plasma membrane components, exemplified by comparisons in diffusion above the nucleus and cytoplasm. To estimate the effect of topography on the diffusion of a protein, a membrane marker can be used, but the marker is a proxy for the membrane area and a given area can be folded in many different ways that in turn alter the transit times. It is not possible to generalize how topography varies in different positions in the cell since both inter and intra cell variations are substantial. Whenever a cell treatment is reported to affect the diffusion of a plasma membrane molecule or the molecule is reported to undergo anomalous diffusion, it needs to be ascertained that changes in the membrane topography are not the underlying cause of the findings.

## Data Availability Statement

The implementation of the simulation, the code to model the FCS recordings as well as the code to compute the autocorrelation is available at https://github.com/RUBION-Nanoscopy/FCS-simulation.

## Author Contributions

AG, SW, PaH, and IP designed and performed the experiments. PaH wrote the simulation and modeling software. AG and PhH performed the simulations. S-GE performed the statistical modeling. All authors analyzed the data. IP and PaH drafted the manuscript and conceived the idea. All authors contributed to the article and approved the submitted version.

## Conflict of Interest

The authors declare that the research was conducted in the absence of any commercial or financial relationships that could be construed as a potential conflict of interest.
