# Supplementary material for: Variations in Plasma Membrane Topography Can Explain Heterogenous Diffusion Coefficients Obtained by Fluorescence Correlation Spectroscopy
Source: Front Cell Dev Biol. 2020 Aug 11;8:767. doi: 10.3389/fcell.2020.00767 (PMC7443568; doi:10.3389/fcell.2020.00767)
Supplement: Supplementary file 1 [file Data_Sheet_1.PDF]

SUPPLEMENTARY MATERIAL

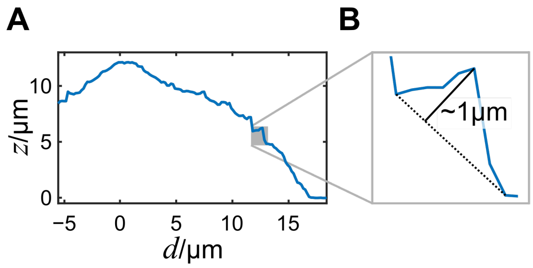

Supplementary Figure 1: Individual topographical features are noticeably larger than the local roughness variations. (A) Height profile along the dashed line as in **Figure 1B**. **(B)** Enlargement of the region marked with the grey rectangle in (A). The height of the protrusion with respect to a linear approximation of the cell silhouette (dotted line) was approximately 1 µm.

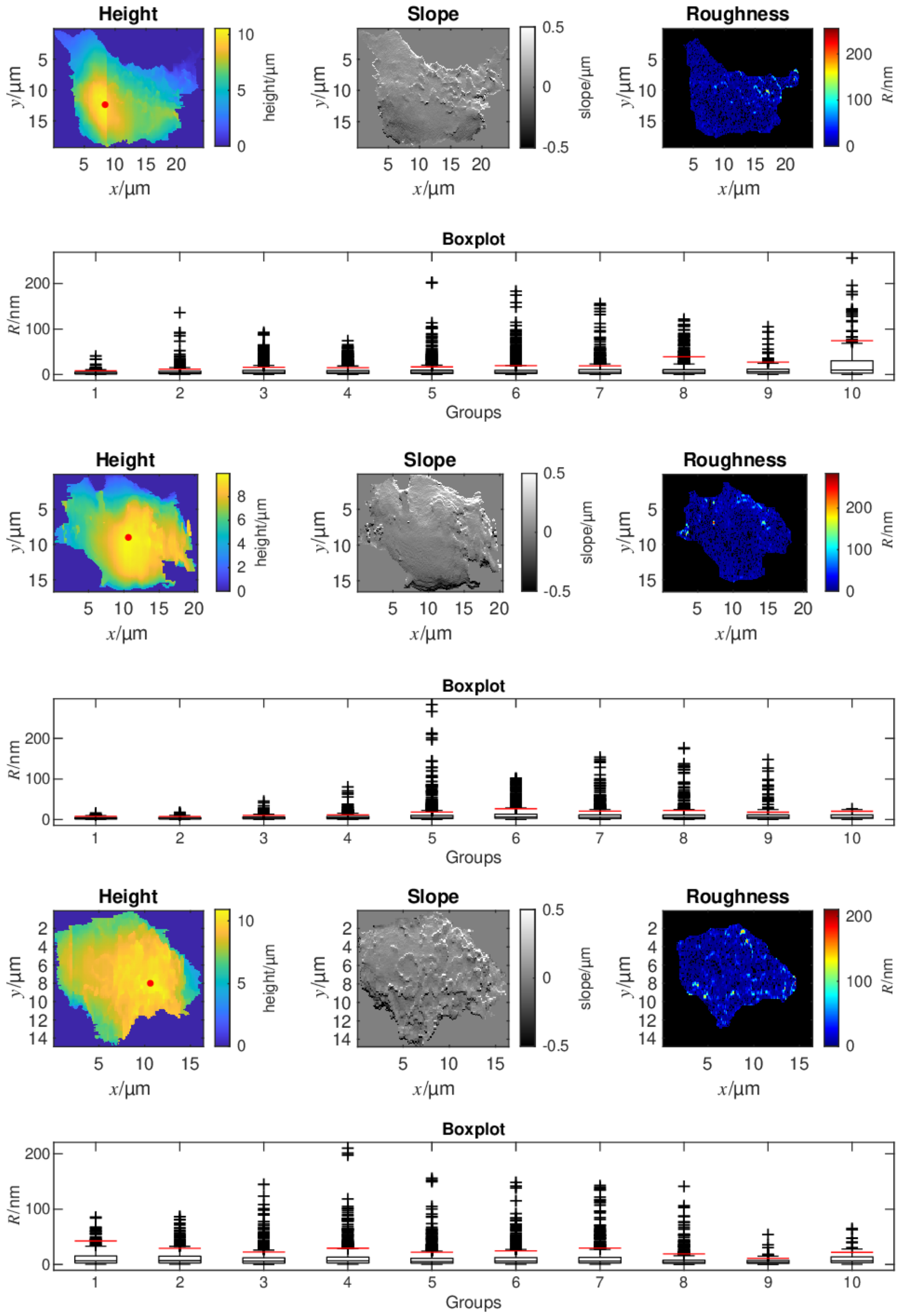

Supplementary Figure 2: The distribution of roughness in all cell samples analysed by SICM. The horizontal red lines in the box plots indicate the 90th percentile of the data within the group.

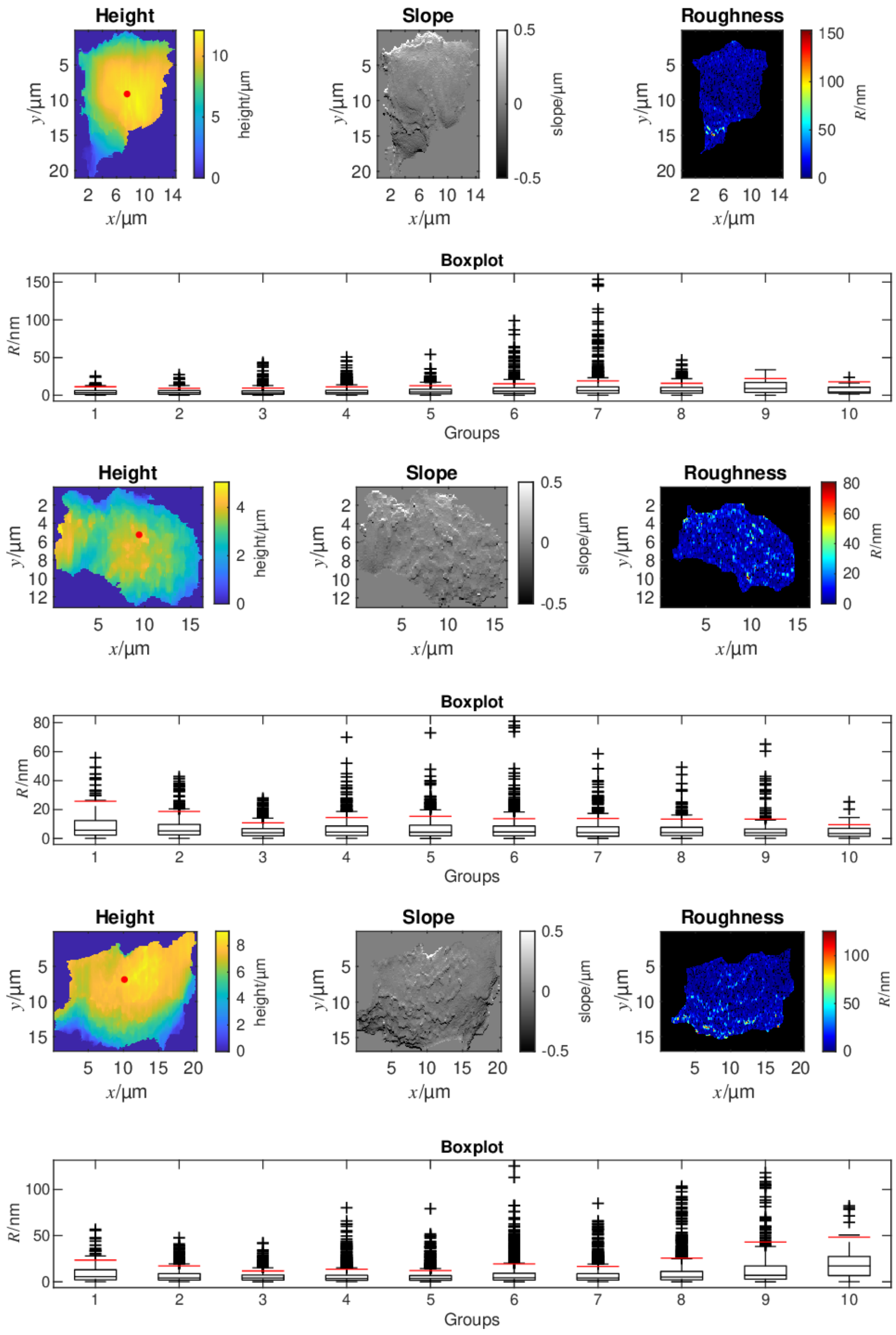

Supplementary Figure 2 (continued)

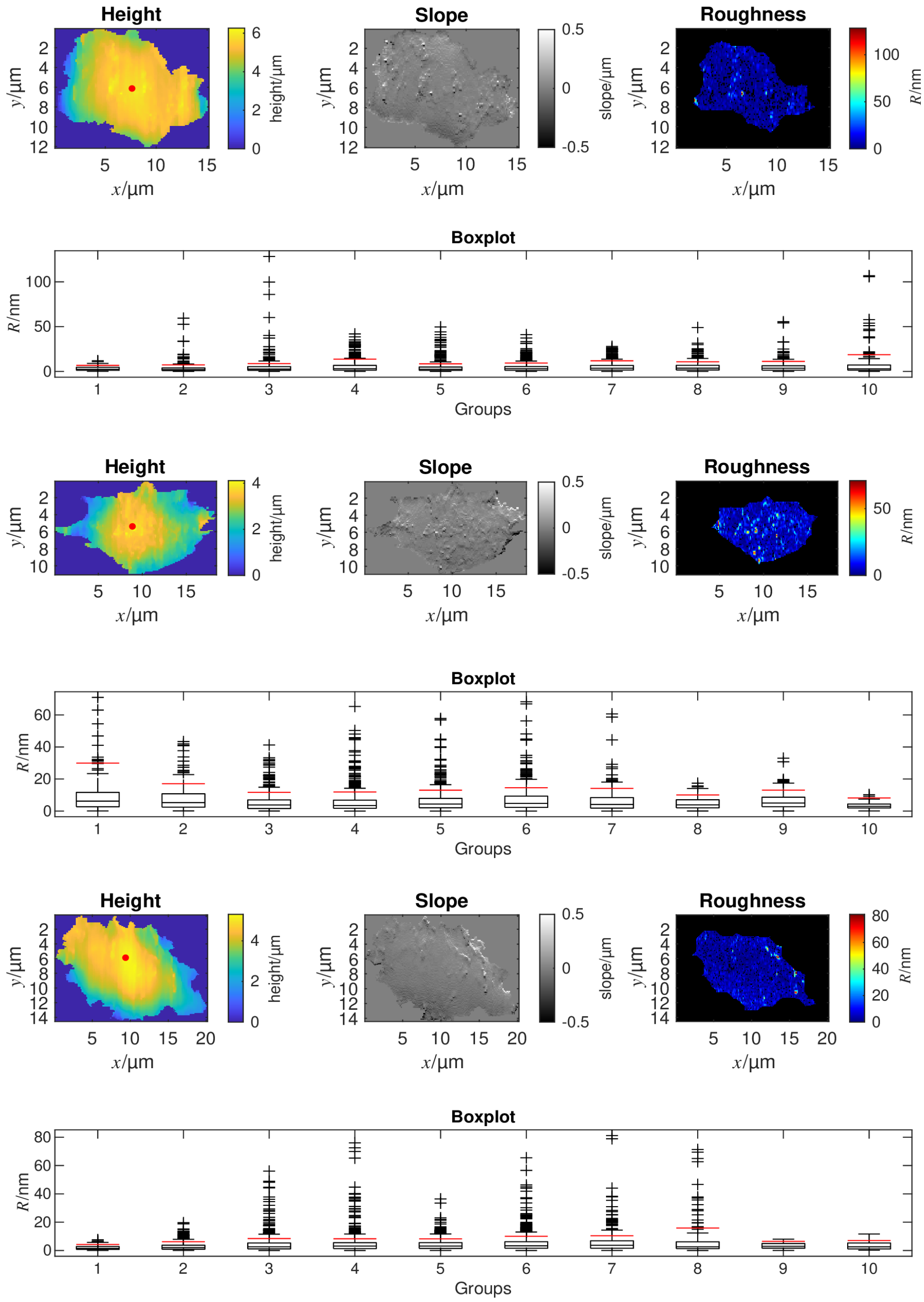

Supplementary Figure 2 (continued)

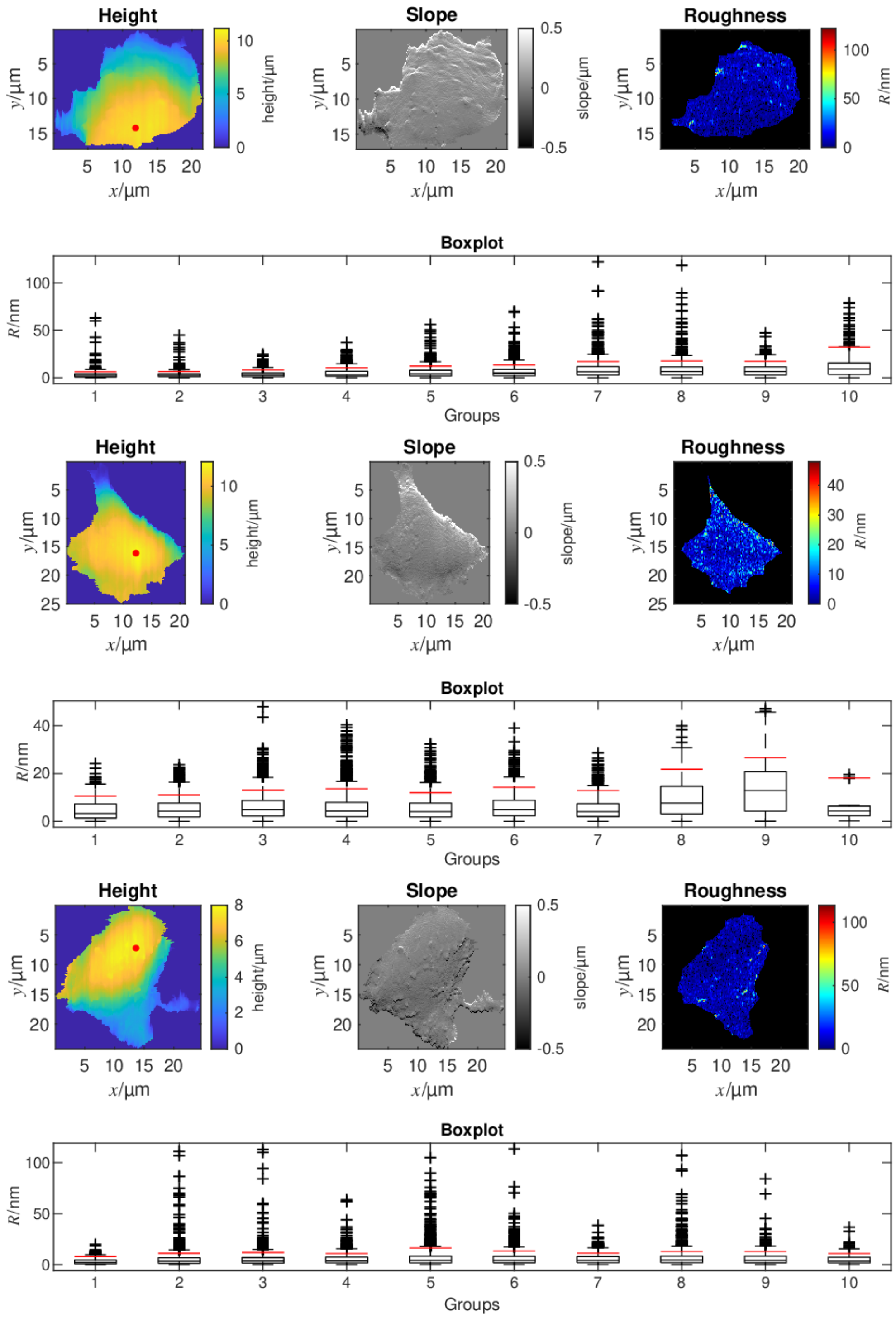

Supplementary Figure 2 (continued)

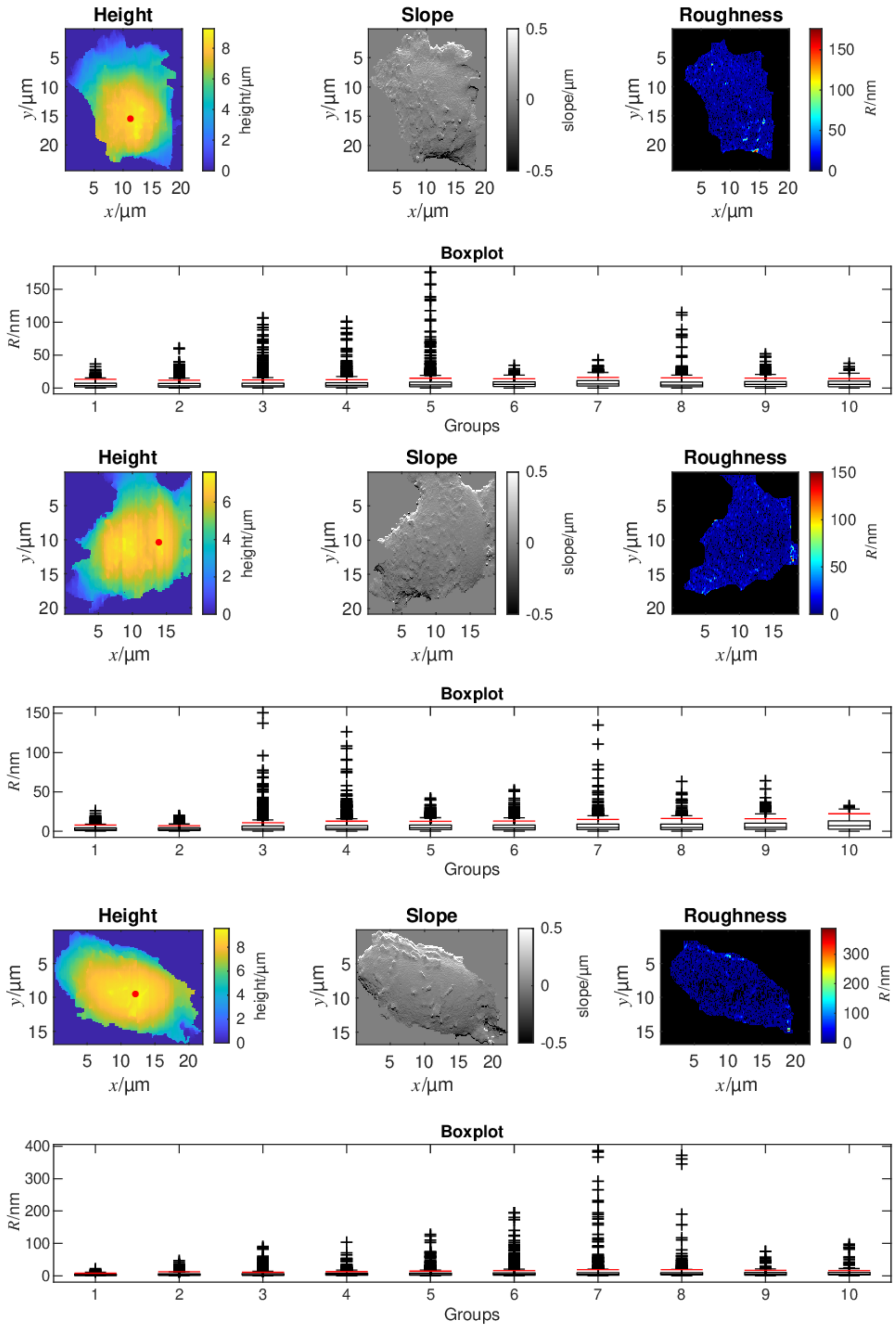

Supplementary Figure 2 (continued)

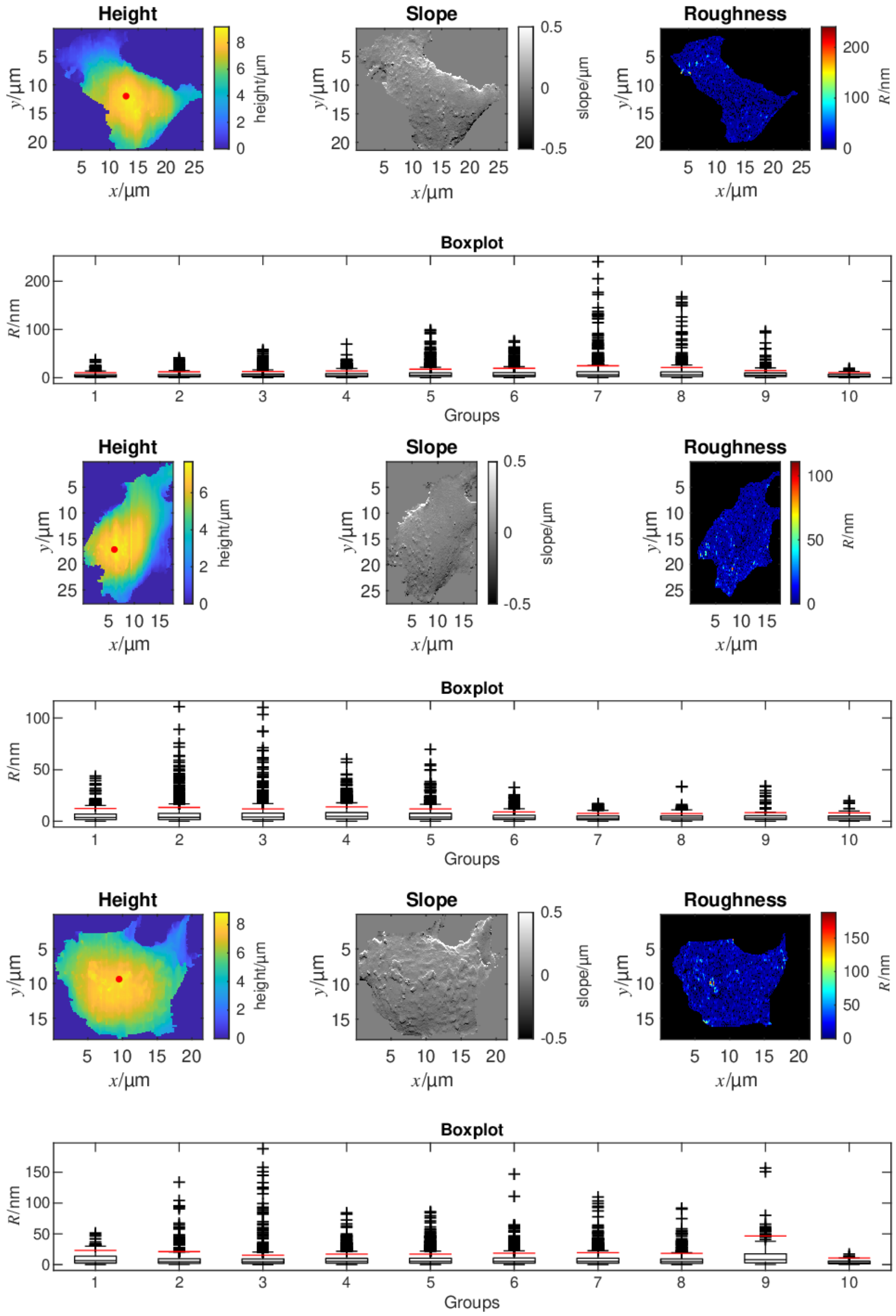

Supplementary Figure 2 (continued)

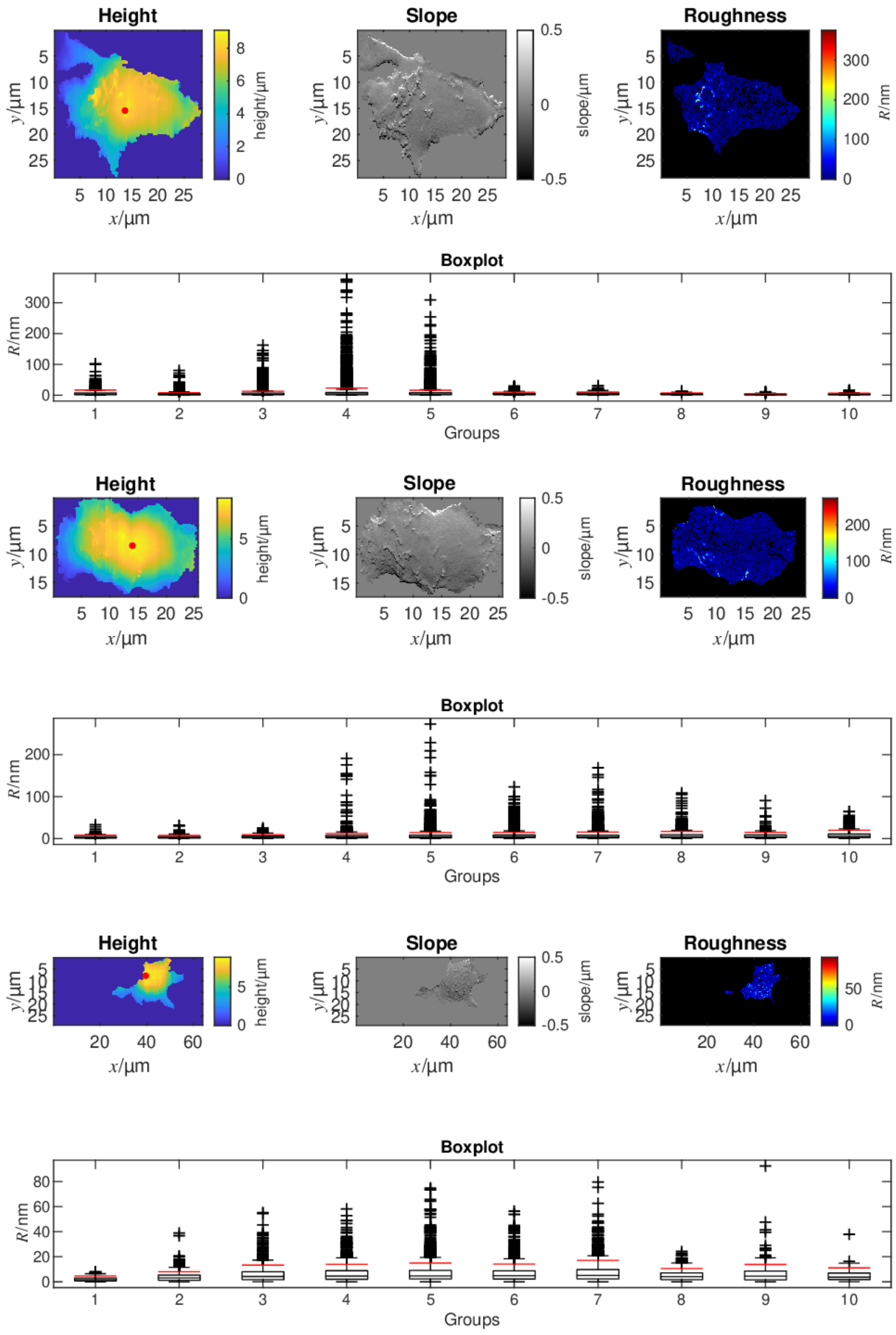

Supplementary Figure 2 (continued)

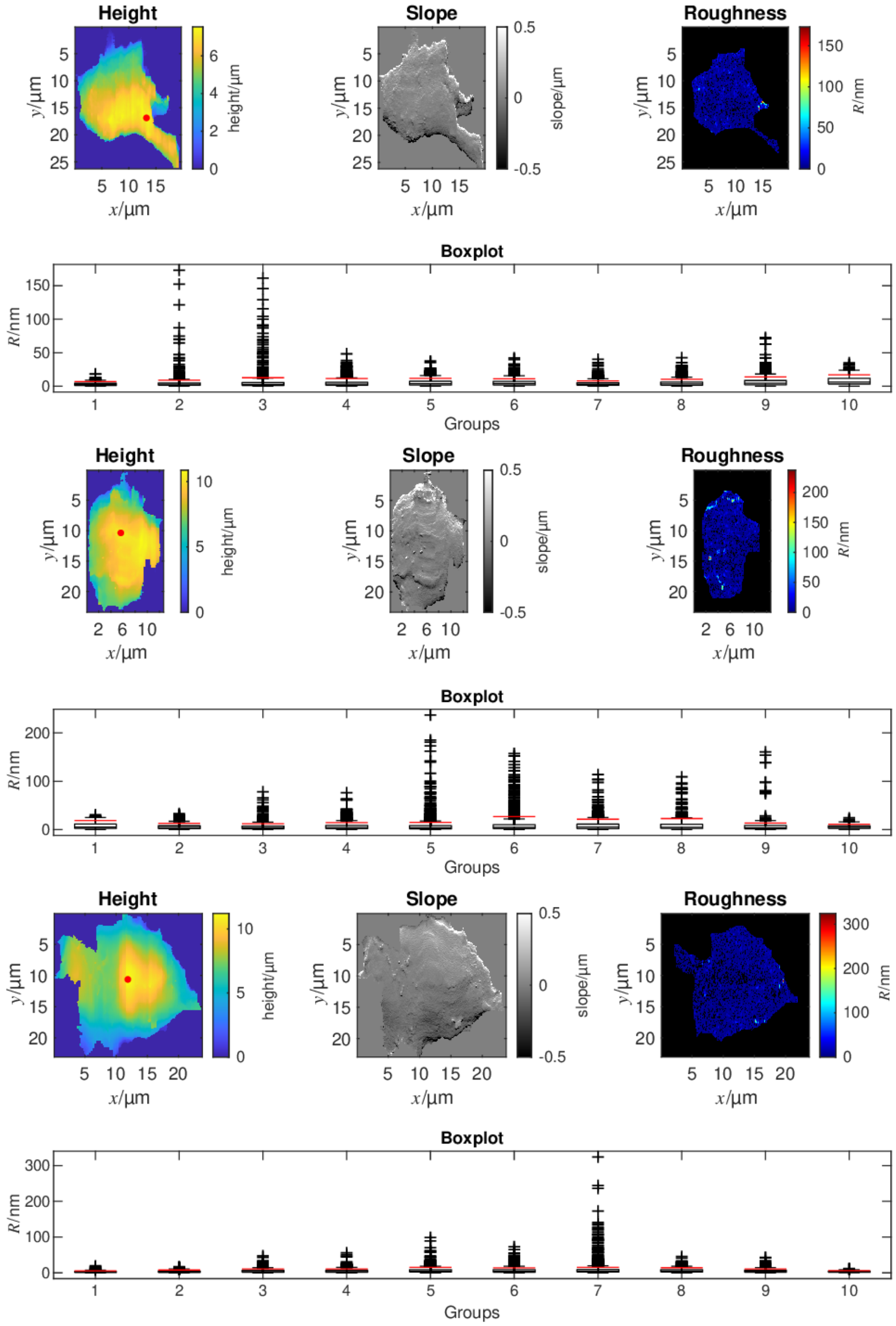

Supplementary Figure 2 (continued)

^

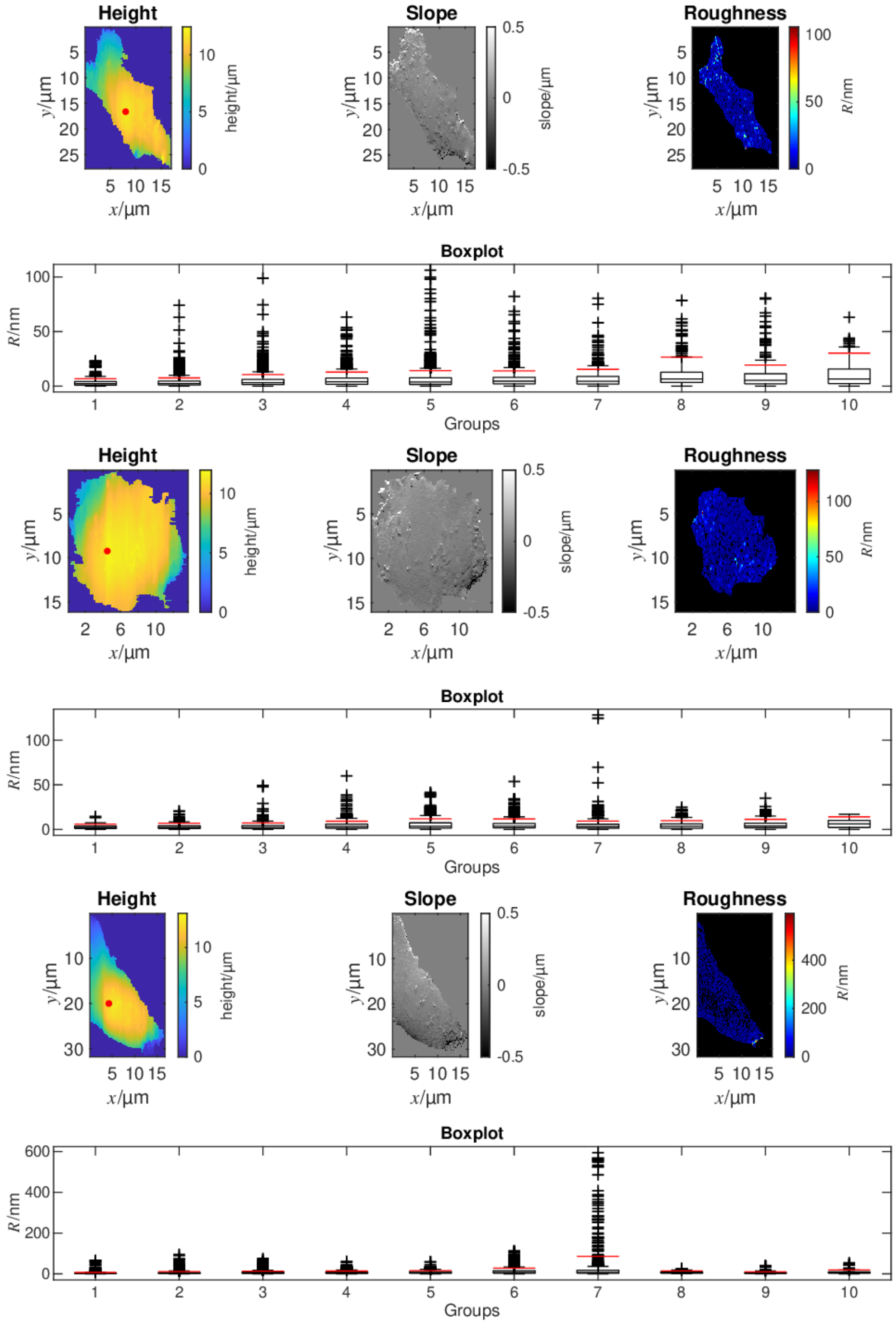

Supplementary Figure 2 (continued)

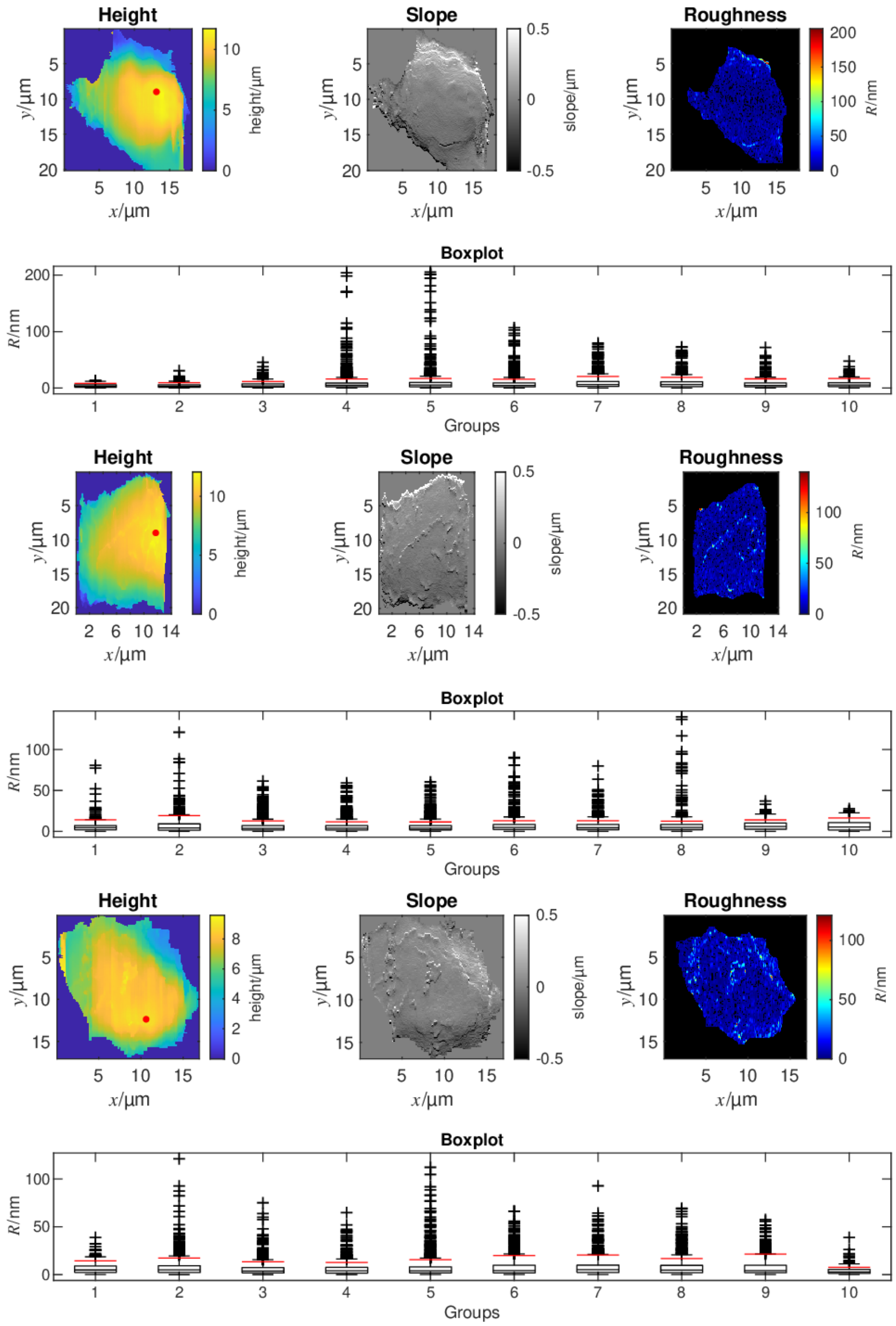

Supplementary Figure 2 (continued)

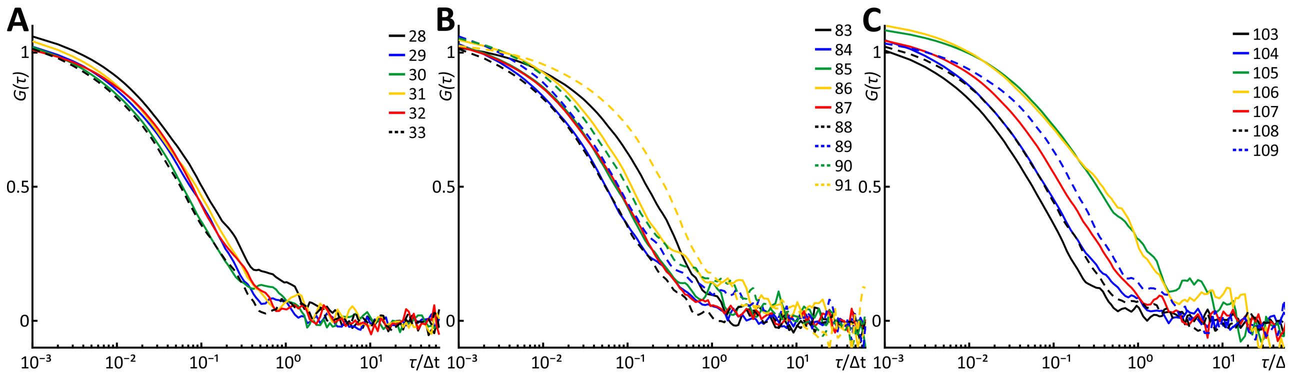

Supplementary Figure 3: FCS-Curves for the data shown in Figure 5.

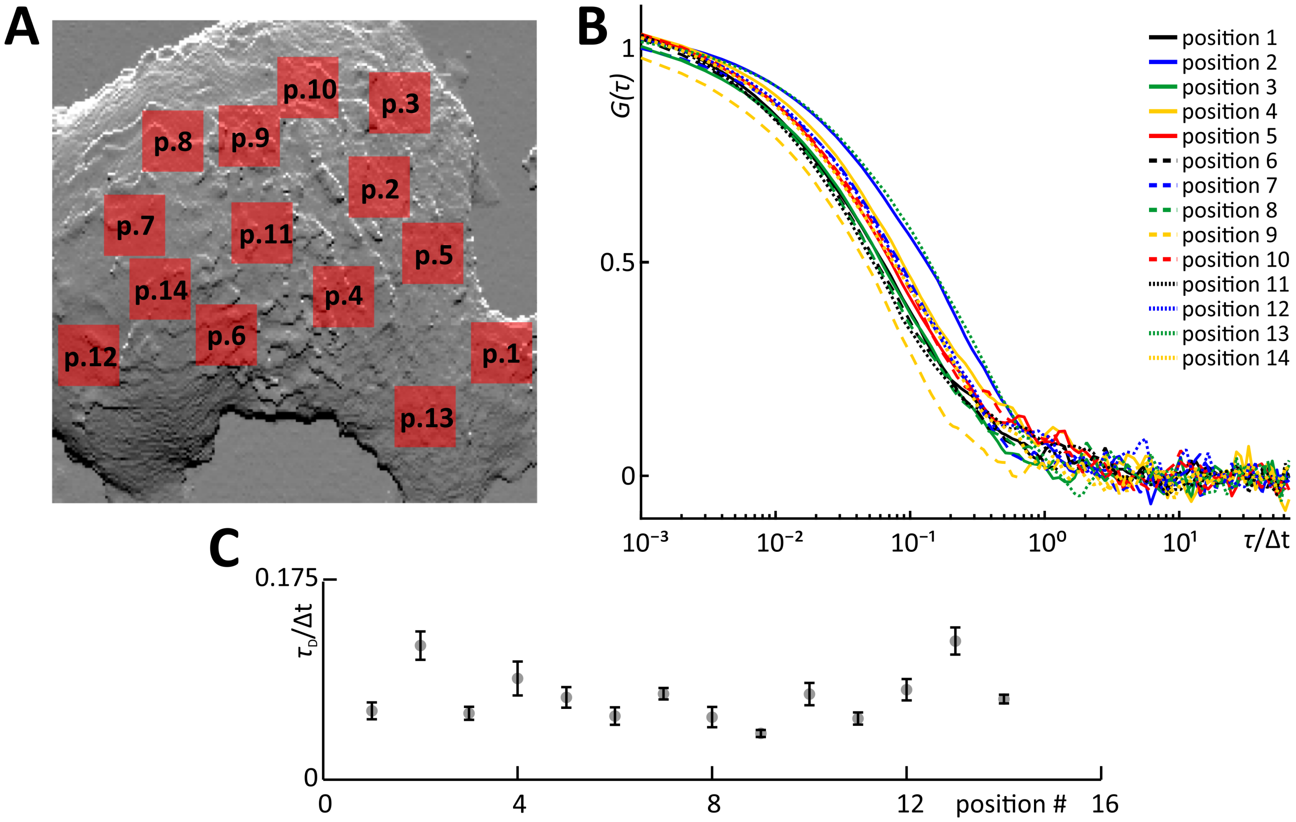

Supplementary Figure 4: Simulated diffusion and modelled FCS recordings at arbitrary positions of the cell shown in Figure 3A and B. **(A)** Overlay of the slope representation of the cell and the membrane sections where diffusion was simulated (red squares). FCS was modelled at the centre of each square. **(B)** The corresponding correlation curves and **(C)** the resulting transit times presented as means±SD, n=5.

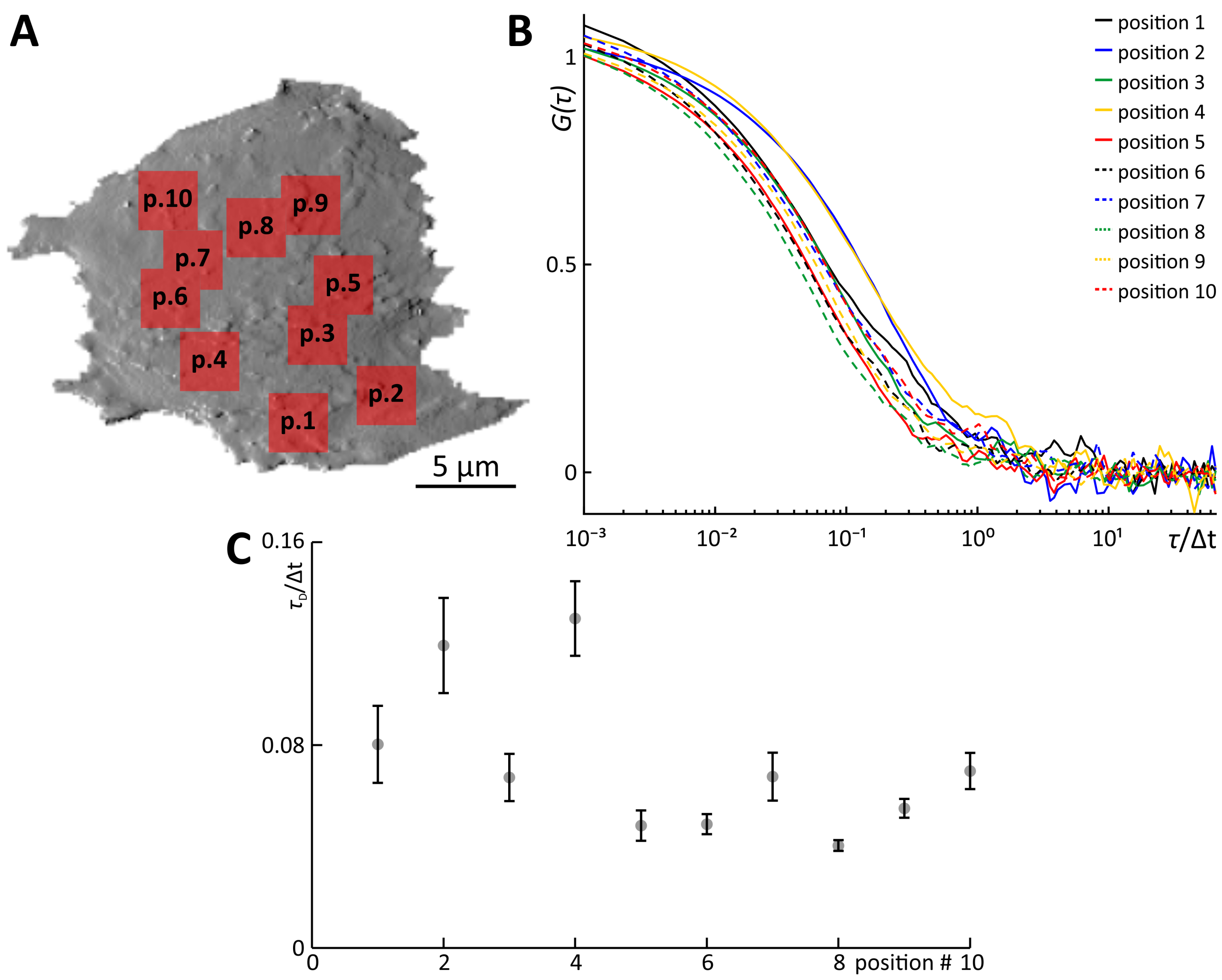

Supplementary Figure 5: Simulated diffusion and modelled FCS recordings at arbitrary positions of a second cell included in the SICM-analysis. (A) Overlay of the slope representation of the cell and the membrane sections where diffusion was simulated (red squares). FCS was modelled at the centre of each square. **(B)** The corresponding correlation curves and **(C)** the resulting transit times presented as means±SD, n=5.

Supplementary Table 1: Adjusted p-values (according to Holm-Bonferroni) for the comparisons shown in Figure 4.

**Group 1 vs.**	**2**	**3**	**4**	**5**	**6**	**7**	**8**	**9**	**10**
p_{adj}	0.5761	0.5377	0.0357	0.0267	0.0090	0.0120	0.0221	0.0357	0.0677
